# Overexpression of *Fkbp11*, a feature of lupus B cells, leads to B cell tolerance breakdown and initiates plasma cell differentiation

**DOI:** 10.1002/iid3.65

**Published:** 2015-06-18

**Authors:** Julie Ruer-Laventie, Léa Simoni, Jean-Nicolas Schickel, Anne Soley, Monique Duval, Anne-Marie Knapp, Luc Marcellin, Delphine Lamon, Anne-Sophie Korganow, Thierry Martin, Jean-Louis Pasquali, Pauline Soulas-Sprauel

**Affiliations:** 1 CNRS UPR3572, Institut de Biologie Moléculaire et Cellulaire, Immunopathologie et Chimie Thérapeutique/Laboratory of Excellence MedalisStrasbourg, F-67084, France; 2 Université de Strasbourg, UFR MédecineStrasbourg, F-67085, France; 3 Department of Anatomopathology, H, ô, pitaux Universitaires de StrasbourgF-67085, France; 4 Department of Clinical Immunology, Hôpitaux Universitaires de StrasbourgF-67085, France; 5 Université de Strasbourg, UFR Sciences PharmaceutiquesIllkirch, F-67401, France

**Keywords:** B cells, *Fkbp11*, lupus, mouse models

## Abstract

Systemic Lupus Erythematosus (SLE) is a severe systemic autoimmune disease, characterized by multi-organ damages, triggered by an autoantibody-mediated inflammation, and with a complex genetic influence. It is today accepted that adult SLE arises from the building up of many subtle gene variations, each one adding a new brick on the SLE susceptibility and contributing to a phenotypic trait to the disease. One of the ways to find these gene variations consists in comprehensive analysis of gene expression variation in a precise cell type, which can constitute a good complementary strategy to genome wide association studies. Using this strategy, and considering the central role of B cells in SLE, we analyzed the B cell transcriptome of quiescent SLE patients, and identified an overexpression of *FKBP11*, coding for a cytoplasmic putative peptidyl-prolyl cis/trans isomerase and chaperone enzyme. To understand the consequences of *FKBP11* overexpression on B cell function and on autoimmunity's development, we created lentiviral transgenic mice reproducing this gene expression variation. We showed that high expression of *Fkbp11* reproduces by itself two phenotypic traits of SLE in mice: breakdown of B cell tolerance against DNA and initiation of plasma cell differentiation by acting upstream of *Pax5* master regulator gene.

## Introduction

Systemic Lupus Erythematosus (SLE) is a severe and prototypic autoimmune disease. The disease is characterized by the production of various pathogenic autoantibodies such as antinuclear antibodies (anti double-stranded DNA, anti-chromatin...). These autoantibodies participate in end-organ damages by a variety of mechanisms, notably via immune complex mediated inflammation, which can result in severe glomerolunephritis and vasculitis. The origin of SLE is generally attributed to a combination of a complex genetic influence 1,2, and badly described environmental factors. In line with this theory, the majority of human SLE occurs in adults and is clinically characterized by a succession of flares interspersed with remission phases. Actual treatments consist in non specific drugs such as nonsteroidal antiinflammatory molecules, antimalarial agents, glucocorticoids, and immunosuppressive drugs 3. A better understanding of the pathology is today necessary to allow for the elaboration of more targeted and less immunosuppressive therapies for SLE, with fewer side effects.

The heterogeneity of SLE is evident from different points of views: the clinical phenotype is different from patient to patient (apart from the almost constant high production of antinuclear autoantibodies), as well as the genetic susceptibility to the disease. Although rare cases of monogenic lupus with a precise phenotype have been described in children (ex. *C1q* deficiency, *TREX1* defects, *SAMHD1* defects) 4, we must consider that adult SLE arises from the building up of many subtle gene variations, each one adding a new brick on the SLE susceptibility, and each one contributing to a phenotypic trait to the disease.

Trying to understand the mechanism of the different phenotypic traits of the disease (loss of immune tolerance leading to autoantibody production, defect of apoptotic debris clearance, immune complexes related kidney pathology, diverse skin manifestations, arthritis…) is a huge and essential effort. On a strategic point of view, one can guess at least two different roads to identify such molecular mechanisms of the SLE phenotypic expressions. The first one starts from the genomic variants already identified during Genome Wide Association Studies (GWAS). GWAS of SLE patients have identified more than 30 genetic polymorphisms that are associated with SLE, but the combination of these variants differs from patient to patient. These SLE susceptibility genes could affect different steps of SLE development including B cell tolerance breakdown leading to autoantibody production (e.g., *PTPN22, BANK1, BLK, LYN*), defective clearance of immune complexes and apoptotic debris (e.g., *ITGAM, Fc gamma R, Complement*), T cell activation (e.g., *PTPN22, CTLA4*) or organ damage (e.g., *Complement, IRF5, TNFAIP3*) 1,2.

However, these variations usually do not identify the causal gene, and it is likely that more than one gene variant is necessary to induce a phenotypic trait (because odds ratio compared to healthy individuals are usually weak).

The second road starts from a comprehensive analysis of gene expression variation in a precise cell type. Indeed, transcriptome analysis can constitute a good complementary strategy to GWAS because it could identify some deregulated genes as a resultant of the biological effects of the diverse genetic variants present in a patient.

This work uses this second strategy, and we have chosen to focus on B cells, because of the central role of this cell type in SLE. Indeed, several lines of evidence indicate that B cells are central to the disease process 5: 1) B cells produce the autoantibodies, some of which are clearly pathogenic by immune complex deposits or by destroying their target; 2) (NZBxNZW)F1 and MRL-Fas^lpr/lpr^ mice (murine models of human SLE) harboring the *xid* mutation, which inactivates Btk and causes a blockade of B cell development and B cell responses, no longer develop lupus phenotype, including autoantibodies and glomerulonephritis 6,7, as do (NZBxNZW)F1 mice having a very restricted IgM transgenic repertoire 8; 3) the disease can be transferred in mice by B cells: immunodeficient SCID (severe combined immunodeficiency) mice populated with pre-B cells of (NZBxNZW)F1 mice develop many of the characteristics of (NZBxNZW)F1 mice, suggesting that genetic defects responsible for the development of SLE disease in (NZBxNZW)F1 mice are expressed in their B cells 9.

In order to better understand the role of B cell gene expression abnormalities in SLE immunopathology, we recently analyzed the B-cell transcriptome of SLE patients focusing on the inactive phase of the disease, to avoid gene variation expression linked to B cell activation which accompanies lupus flares 10. We started to create new mouse models to reproduce the human SLE gene expression variations and have already shown that this functional genomic approach is successful with *Carabin gene* 11. One of these genes, *FKBP11*, is overexpressed in sorted B cells of SLE patients in our analysis. *FKBP11* gene encodes the FKBP19 protein, a member of the peptidyl-prolyl *cis/trans* isomerase (PPIase) FKBP family. The FKBP19 protein is a FK506 binding protein, containing a N-terminal signal sequence, a PPIase domain, a putative transmembrane domain, and lacking a calcium-binding EF-hand (helix-loop-helix structural domain), which is typical of several FKBP members of the secretory pathway. Notably, it is expressed in lymphoid tissue, in particular during plasma cell differentiation, but its precise biological role in B cells is unknown 12. Thus, to understand the biological significance of the overexpression of *FKBP11* in B cells during human SLE, we created lentiviral transgenic mice reproducing the high level expression of *FKBP11*. The results show that variation in the expression of this single gene is able to induce two phenotypic traits of human lupus: B cell tolerance breakdown and initiation of plasma cell differentiation. In addition, our results add new important informations on the role of *Fkbp11* in B cell physiology.

## Results

### Overexpression of *FKBP11* in a subset of quiescent SLE patients

**Figure 1:**
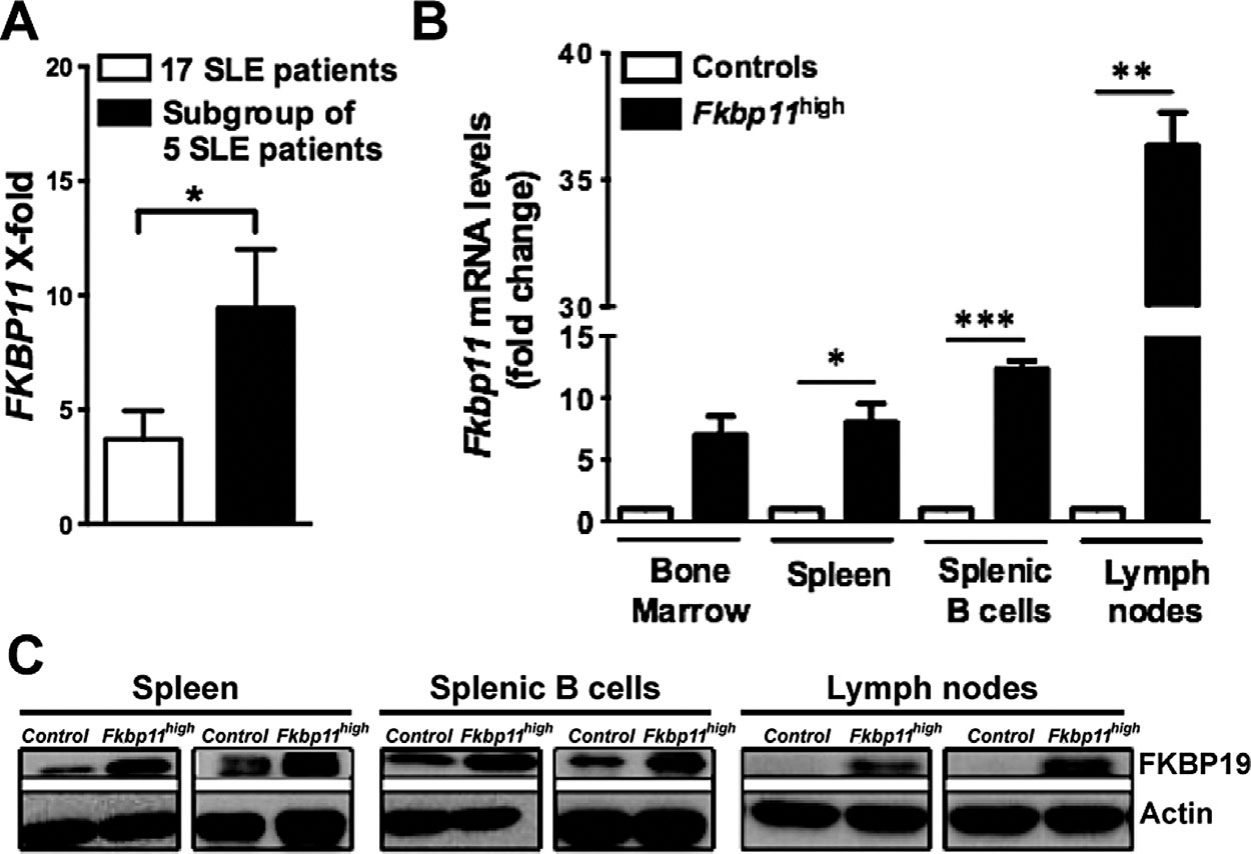
*FKBP11* is overexpressed in B cells from quiescent SLE patients. This overexpression was reproduced in *Fkbp11*^high^ lentiviral transgenic mice. (A) *FKBP11* mRNA expression levels in transcriptome analysis of purified B cells from 17 SLE quiescent patients (white bar), and from a subgroup of 5 SLE quiescent patients (black bar) compared to 9 healthy age and sex-matched controls. X-fold represents the 2^exp(Pi-Tmean)^ value for the patients, where Pi is the value of the *FKBP11* probe set signal for a given patient, and T_mean_, the mean value of signals for the same probe set for the controls. (T_mean_ = 6.096) (Error bars, SEM; *P < 0.05, Mann & Whitney test). (B) Quantitative real time RT-PCR analysis of *Fkbp11* mRNA expression in total bone marrow cells, splenocytes, purified splenic B cells and total lymph node cells from 3-month-old *Fkbp11*^high^ (n = 3) and littermate control mice (n = 3). Each sample was normalized to the endogenous control *Hprt1*. Each bar represents the level of *Fkbp11* mRNA relative to control mice, for each condition (*P < 0.05; **P < 0.005; ***P < 0.0005: unpaired *t* test with Welch correction). (Error bars, SEM). (C) Western blot analysis of FKBP19 expression in splenocytes, purified splenic B cells and total lymph node cells from *Fkbp11*^high^ and littermate control mice. Actin was used as loading control.

We recently analyzed a pangenomic transcriptome of purified CD19^+^ peripheral B cells in patients with inactive SLE in comparison to B cells from age- and sex- matched controls 10. *FKBP11* was overexpressed in all patients with a strong statistical significance using two different probes in the DNA microarrays (*p* = 0.006 for the *FKBP11* probeset 1, and *p* = 0.0113 for the *FKBP11* probeset 2, see reference 10 and also GEO Series accession number GSE30153 (http://www.ncbi.nlm.nih.gov/geo/query/acc.cgi?acc=GSE30153)). Based on probeset 1, *FKBP11* was overexpressed in all patients with a mean increase of 4-fold relative to controls. The overexpression of *FKBP11* was much higher (mean of 9-fold over healthy controls) in a subset of five patients (Fig. 1A) displaying a distinct gene expression with many genes implicated in the Unfolded Protein Response (UPR) 10. *FKBP11* overexpression was validated by quantitative real-time RT-PCR in patients versus controls 10. The role of *FKBP11* in B cells and in autoimmunity has never been described. These data led us to investigate in detail the possible role of *FKBP11* overexpression in promoting SLE.

### Production of lentiviral transgenic mice overexpressing *Fkbp11*

In order to understand the possible consequences of *FKBP11* overexpression on B cell function and autoimmunity, we developed a functional genomic approach consisting in the production and analysis of a new lentigenic mouse model overexpressing *Fkbp11*, on C57BL/6 background.

The pTRIP lentiviral vector used for the production of *Fkbp11* transgenic mice (Supplementary Fig. S1, A and B) allows for the separated and equal expression of GFP reporter and of murine FKBP19 (protein encoded by *Fkbp11* gene), thanks to a T2A peptide derived from picornavirus 13. The expression of proteins is under the control of the human Ubiquitin C (UbiC) promoter allowing a good expression of the gene of interest in lymphoid organs, notably in B cells 14,15. We selected a founder with a single proviral integration for further studies, which was named *Fkbp11*^high^(Supplementary Fig S1C). Using the same strategy, we also produced a control transgenic mouse line (GFP^+^ control line), with a pTRIP lentiviral vector resulting in the expression of GFP alone, from one unique proviral integration site (Supplementary Fig. S1, D and E).

We first evaluated the expression of *Fkbp11* mRNA in lymphoid organs in *Fkbp11*^high^ transgenic mice, by quantitative real-time RT-PCR. *Fkbp11* is overexpressed in total bone marrow cells, splenocytes, purified splenic B cells and total lymph node cells (Fig. 1B). Western blot analysis confirmed the overexpression of FKBP19 in splenocytes, splenic sorted B cells and lymph node cells in *Fkbp11*^high^ mice (Fig. 1C), but FKBP19 expression was barely detectable in bone marrow cells. GFP^+^ control mice, while expressing GFP in splenocytes, splenic B cells and lymph node cells, did not overexpress *Fkbp11* mRNA nor FKBP19 protein (Supplementary Fig. S1F and G) in lymphoid organs, arguing against a transgene-dependent effect for *Fkbp11* overexpression in *Fkbp11*^high^ mouse line.

### Lymphoid hyperplasia and increased T-independent B cell response in *Fkbp11*^high^ mice

In order to analyze the consequences of *Fkbp11* overexpression *in vivo*, we studied the immunological phenotype of 8-month-old *Fkbp11*^high^ mice. As shown in Table1, subset proportions were not different between GFP^+^ control transgenic mice and GFP**^-^** (C57BL/6) littermate control mice. Therefore GFP^−^ control mice were used for the rest of the experiments. The control mice used in this study are always littermates for the transgene-positive counterparts.

**Table 1:** B and T cell phenotypes in *Fkbp11*^high^ and GFP^+^ control mice, compared to littermate control mice

	Littermate Controls	*Fkbp11*^high^	GFP^+^ Controls
Total cellularity			
Bone Marrow	28 10^6 ^± 0,8	45 10^6 ^± 0,4^*^^*^^*^	N.D.
Spleen	60.10^6 ^± 17	82 10^6 ^± 22^*^^*^	52 10^6 ^± 19
Lymph Nodes	3 10^6 ^± 1,5	5.3 10^6 ^± 3,5^*^	3.2 10^6 ^± 0,4
Bone Marrow	n = 6	n = 5	
Total B cells	1.8 10^6 ^± 0.5	3 10^6 ^± 0.7^*^^*^^*^	N.D.
Pro-Pre B	1.4 10^6 ^± 0.3	3.2 10^6 ^± 0.7^*^	N.D.
Immature	0.8 10^6 ^± 0.3	1.1 10^6 ^± 0.2	N.D.
Transitional	0.3 10^6 ^± 0.1	0.5 10^6 ^± 0.2^*^	N.D.
Recirculating mature	0.7 10^6 ^± 0.2	1.1 10^6 ^± 0.4^*^	N.D.
Spleen	n = 15	n = 14	n = 4
Total B cells	27 10^6 ^± 8	38 10^6 ^± 10 ^*^^*^	26 10^6 ^± 10
T1	3.5 10^6 ^± 1.3	4.9 10^6 ^± 1.9^*^^*^	3.8 10^6 ^± 2
T2	1.2 10^6 ^± 0.6	1.9 10^6 ^± 1	1.6 10^6 ^± 0,5
Follicular	18.5 10^6 ^± 5	25 10^6 ^± 8^*^	17 10^6 ^± 7
MZ	1.4 10^6 ^± 0.7	1.7 10^6 ^± 0.9	1,4.10^6 ^± 0,7
CD4^+^ T cells	13 10^6 ^± 3	16 10^6 ^± 4	10 10^6 ^± 4
CD8^+^ T cells	9 10^6 ^± 3	12 10^6 ^± 3^*^	7 10^6 ^± 2
Lymph Nodes	n = 12	n = 12	n = 4
Total B cells	0.8 10^6 ^± 0.4	1.4 10^6 ^± 0.6^*^^*^	0.8 10^6 ^± 0.1
CD4^+^ T cells	0.9 10^6 ^± 0.6	2.2 10^6 ^± 1.5^*^	1.1 10^6 ^± 0.1
CD8^+^ T cells	1 10^6 ^± 0.5	2.6 10^6 ^± 2.1^*^	1 10^6 ^± 0.2

**Figure 2:**
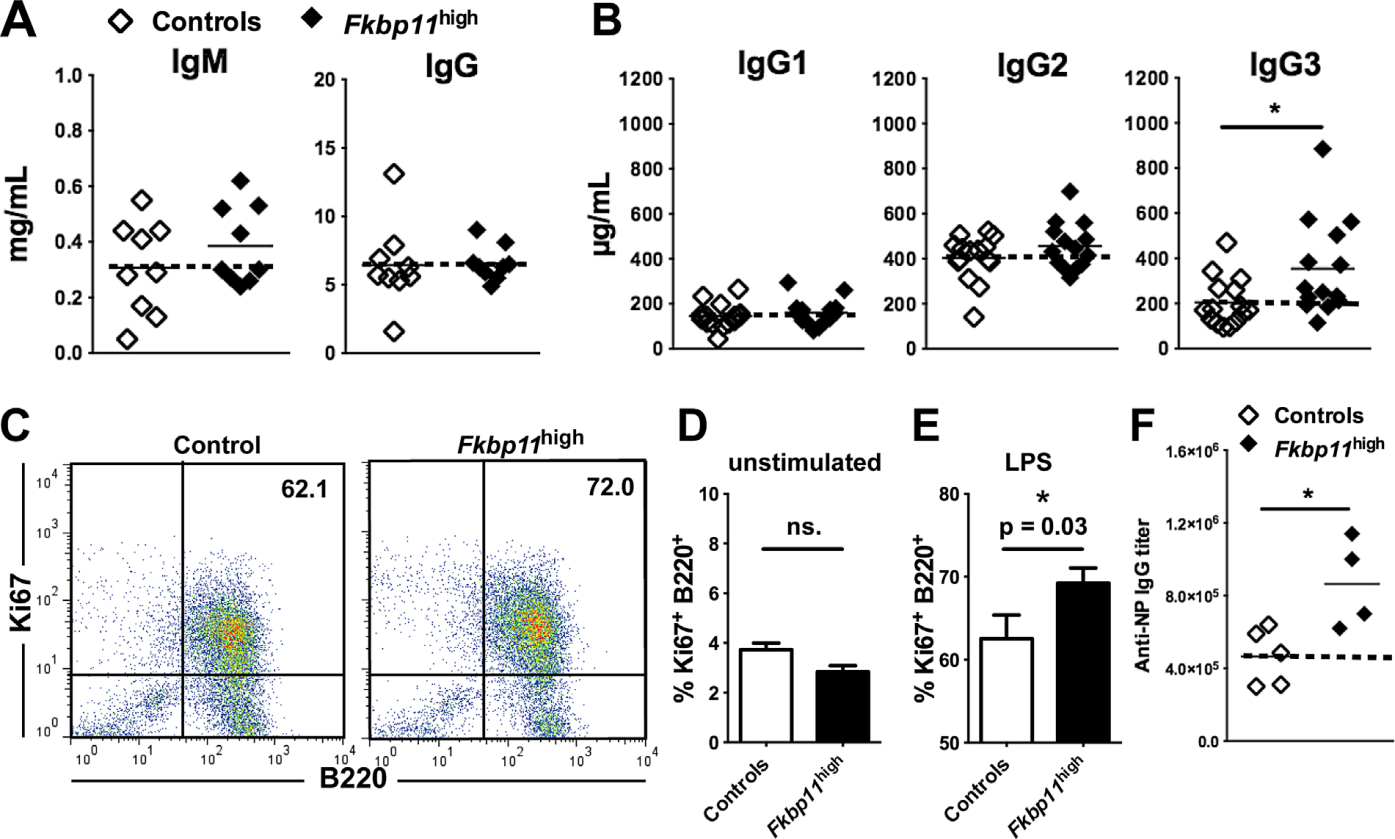
Basal and antigen-induced serum Ig production in *Fkbp11*^high^ mice. (A to E) Sera from 8-month-old *Fkbp11*^high^ and littermate control mice (controls) were collected and total IgM and IgG (A), IgG1, IgG2b and IgG3 (B) were determined by ELISA. Each point represents the result for one animal. (C) Flow cytometry analysis of B cell proliferation after stimulation of splenocytes with LPS for 3 days *in vitro*. A representative FACS plot with percentages of B220^+^Ki-67^+^ of stimulated cells (LPS) from 8-month-old *Fkbp11*^high^ and littermate control mice (controls) is shown. (D, E) Percentages of B220^+^Ki-67^+^ of unstimulated cells (D) and stimulated cells (LPS) (E) from 8-month-old *Fkbp11*^high^ and littermate control mice (controls) are shown (*n*=10 for *Fkbp11*^high^, n = 8 for control mice). (statistics: Mann & Whitney test; ns.: non significant). There was no difference in cell survival in unstimulated and LPS-stimulated cells between the two groups of mice, based on propidium iodide staining. (F) 6- to 8-week-old *Fkbp11*^high^ and littermate control mice were injected intraperitoneally with 100μg of NP-LPS at days 0, 10 and 20. Anti-NP IgG titers were determined at day 30 by ELISA. (*P < 0.05, Mann & Whitney test). The dotted lines indicate the means for control groups. There was no difference in anti-NP IgG titers at day 10 and day 20.

Interestingly, *Fkbp11*^high^ mice developed lymphoid hyperplasia, characterized by an increase of bone marrow, spleen and lymph node total cellularity. This increase occurred in all characterized B and T-cell subpopulations, but was globally more pronounced for B cells (Table1). Compared to control mice, basal activation of B cells and T cells was not modified in *Fkbp11*^high^ mice, as measured by the *ex vivo* expression of activation markers (Supplementary Fig. S2). In addition, at baseline, the production of serum IgG3 was increased in *Fkbp11*^high^ mice in a statistically significant manner, compared to control mice, although the levels of serum IgM, IgG, IgG1, and IgG2b was not different (Fig. 2, A and B).

In concordance with the *in vivo* lymphoid hyperplasia, B cell proliferation was increased after stimulation of splenocytes from *Fkbp11*^high^ mice with LPS for 3 days *in vitro*, compared to splenocytes from littermate control mice (Fig. 2, C–E). Finally, to test B cell responses *in vivo*, we immunized *Fkbp11*^high^ and littermate control mice with T-dependent (ovalbumin, OVA) and T-independent (immunization with 4-hydroxy-3-nitrophenylacetyl (NP)-LPS) antigens. The total IgG, and IgG1 immune response against OVA was unaffected by *Fkbp11* overexpression (data not shown). However, compared to control mice, anti NP-LPS IgG immune response was amplified in *Fkbp11*^high^ mice compared to control mice (Fig. 2F).

Together, these data show that *Fkbp11* overexpression led to lymphoid hyperplasia and increased T-independent B cell responsiveness (higher IgG3 at baseline and more robust anti-NP-LPS IgG after immunization with NP-LPS) *in vivo*.

### *Fkbp11* overexpression led to B cell tolerance breakdown

**Figure 3:**
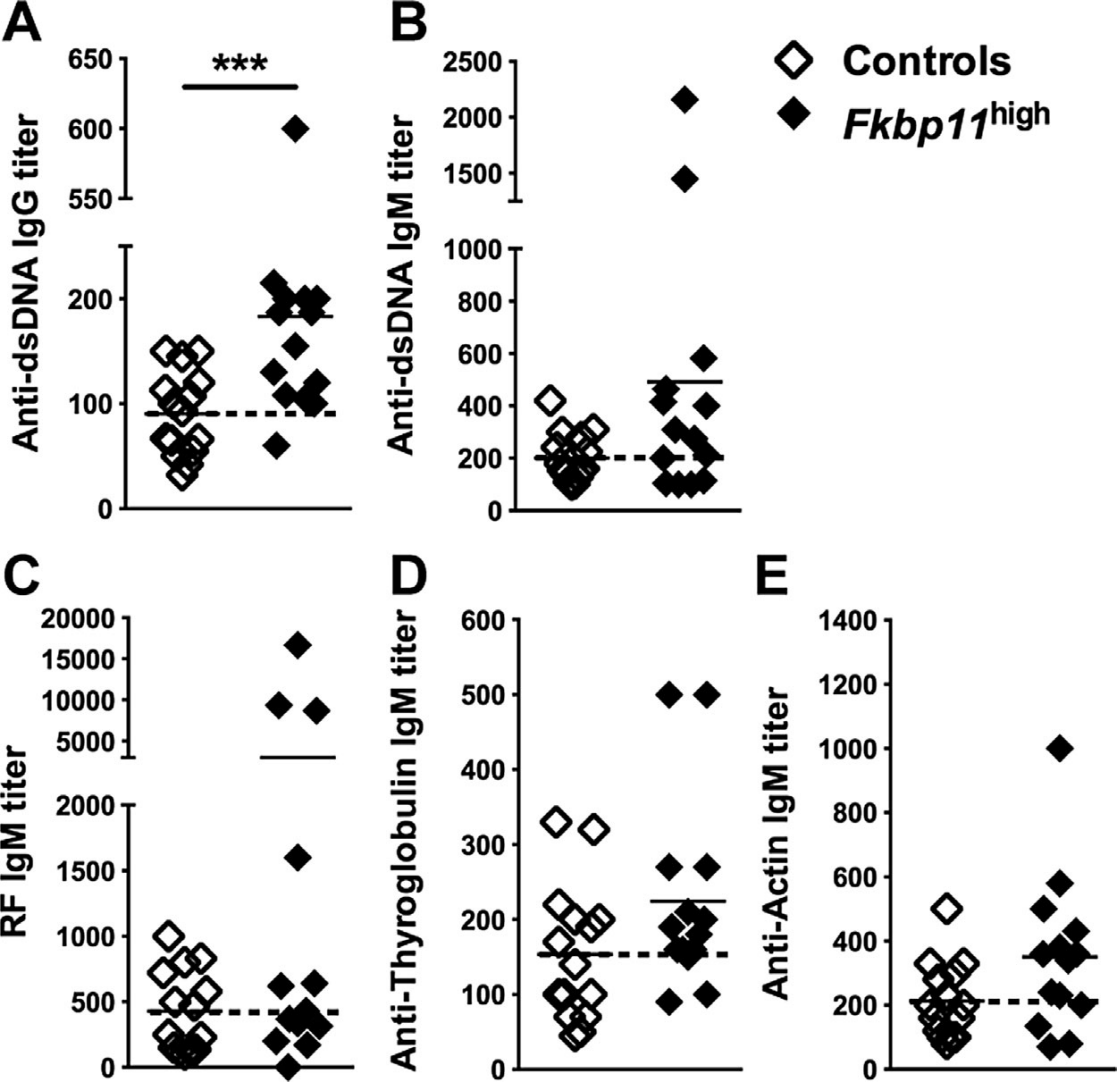
Breakdown of B cell tolerance in *Fkbp11*^high^ mice. (A to E) Anti-dsDNA IgG (A), anti-dsDNA IgM (B), RF IgM (C), anti-thyroglobulin IgM (D), and anti-actin IgM (E) titers were determined by ELISA in sera of 8-month-old *Fkbp11*^high^ and littermate control mice, by ELISA. Each point represents the result for one animal. (***P < 0.005, Mann & Whitney test). The dotted lines indicate the means for control groups.

Since we initially observed *Fkbp11* overexpression in human SLE B cells, it was of importance to test for the presence of autoantibody production in *Fkbp11*^high^ mice. The transgenic mice produced more IgG anti-double stranded (ds) -DNA antibodies than control mice (Fig. 3A). Not only these anti-dsDNA antibodies were produced, but some of the transgenic animals also made more IgM autoantibodies as Rheumatoid Factors (31% of *Fkbp11*^high^ mice produced more than the mean value + standard deviation of controls), anti-thyroglobulin (29%) and anti-actin (57%) (Fig. 3, B–E), indicating a more general breakdown in B cell tolerance in these mice. However, this breakdown of B cell tolerance did not lead to the development of autoimmune symptoms, including glomerulonephritis, as assessed by proteinuria and immunohistological analysis of the kidneys (data not shown, n = 14 mice analyzed in each group).

**Figure 4:**
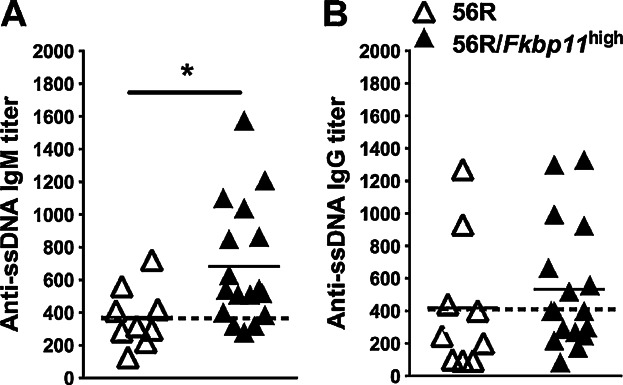
*Fkbp11* overexpression on 56R background breaks B cell tolerance against ssDNA. (A, B) Sera from 12-month-old 56R *Fkbp11*^high^ and 56R littermate control mice were collected then titers of anti-ssDNA IgM (A) and IgG (B) were determined by ELISA. Each point represents the result for one animal. (*P < 0.05: Mann & Whitney test). The dotted lines indicate the means for control groups.

To gain insight into the breakdown of B cell tolerance in *Fkbp11*^high^ mice, we crossed them with the 56R anti-DNA heavy chain transgenic mouse model on C57BL/6 background. The 56R anti-DNA transgenic model (whose B cells express a transgenic heavy chain, associated with a restricted repertoire of endogenous light chains) was shown to produce mainly IgM anti-single-stranded (ss) -DNA 16,17. Twelve-month-old 56R/*Fkbp11*^high^ mice produced significantly more IgM anti-ssDNA autoantibodies than control 56R animals, confirming that the overexpression of *Fkbp11* promoted the loss of B cell tolerance (Fig. 4, A and B).

Considering what is described in the 56R model, this result could be linked to at least three distinct mechanisms: abnormal central autoreactive B cell deletion, defective B cell anergy or defective B cell receptor editing. We did not find any experimental evidence for the first hypothesis since the numbers of IgM^a+^ (transgenic heavy chain) B cells in the bone marrow (Supplementary Fig. S3A) and spleen (Supplementary Fig. S3B), as well as the ratios of IgM^a+^ and IgM^b+^ (endogenous heavy chain) B cells in spleen (data not shown), are not modified by the high expression of *Fkbp11*. To test for the second scenario, we analyzed 56R/*Fkbp11*^high^ mice for the level of surface IgM^a^ expression, and for the B cell response to *in vitro* activation. We did not observe any downregulation of the basal surface IgM^a^ expression on B lymphocytes (Supplementary Fig. S3C). In addition, stimulation of B cells from 56R/*Fkbp11*^high^ mice with BCR-dependent (anti-IgM antibody, or CG-50, used to specifically stimulate anti-DNA B cells 18,19, or CG-Neg as a negative control 19), or BCR-independent stimulus (LPS), did not lead to higher overexpression of activation markers (CD86, CD44, MHCII), to increased proliferation, nor to increased secretion of IgM anti-ssDNA and anti-dsDNA (Supplementary Fig. S3, D–F), compared to B cells from 56R mice. In conclusion, the breakdown of B cell tolerance in 56R/*Fkbp11*^high^ mice could not be explained by a measurable defect in B cell deletion or anergy. Therefore, we wondered if these findings could be explained by a defect of receptor editing. To test this hypothesis, RS rearrangements, which constitute a marker of light chain receptor editing 20, have been analyzed in sorted splenic transitional T1 (B220^+^/IgM^a+^/CD21^−^/CD23^−^), marginal zone (B220^+^/IgM^a+^/CD21^high^/CD23^−^), and follicular (B220^+^/IgM^a+^/CD21^low^/CD23^+^) 21 autoreactive IgM^a+^ cells from 56R/*Fkbp11*^high^ mice, compared to 56R mice. We did not observe a significant difference in RS rearrangement levels in each of the IgM^a+^ autoreactive B cell subpopulations, in 56R/*Fkbp11*^high^ mice compared to 56R mice (Supplementary Fig. S3G). Finally, we sequenced the Vκ genes in T1 population and found a similar distribution of Vκ families between 56R/*Fkbp11*^high^ and 56R mice, although with a greater usage of the minor Vκ1 and Vκ5 families (Supplementary Fig. 3H). However, we do not know if the usage of these minor families is linked to the anti-DNA activity. Furthermore, we did not find any difference in Jκ usage between the two groups of mice (data not shown).

In conclusion, *Fkbp11* overexpression facilitates B cell tolerance breakdown by a mechanism which is not known yet. These results may be linked to the variation of expression of *Fkbp11*, which is tightly regulated during B-cell maturation. Indeed, *Fkbp11* expression is increased in bone marrow transitional T1 B cells where central tolerance is known to take place (Supplementary Fig. S4, A and B).

### *Fkbp11* overexpression exacerbated some traits of the immunopathology of B6*^lpr/lpr^* mice

**Figure 5:**
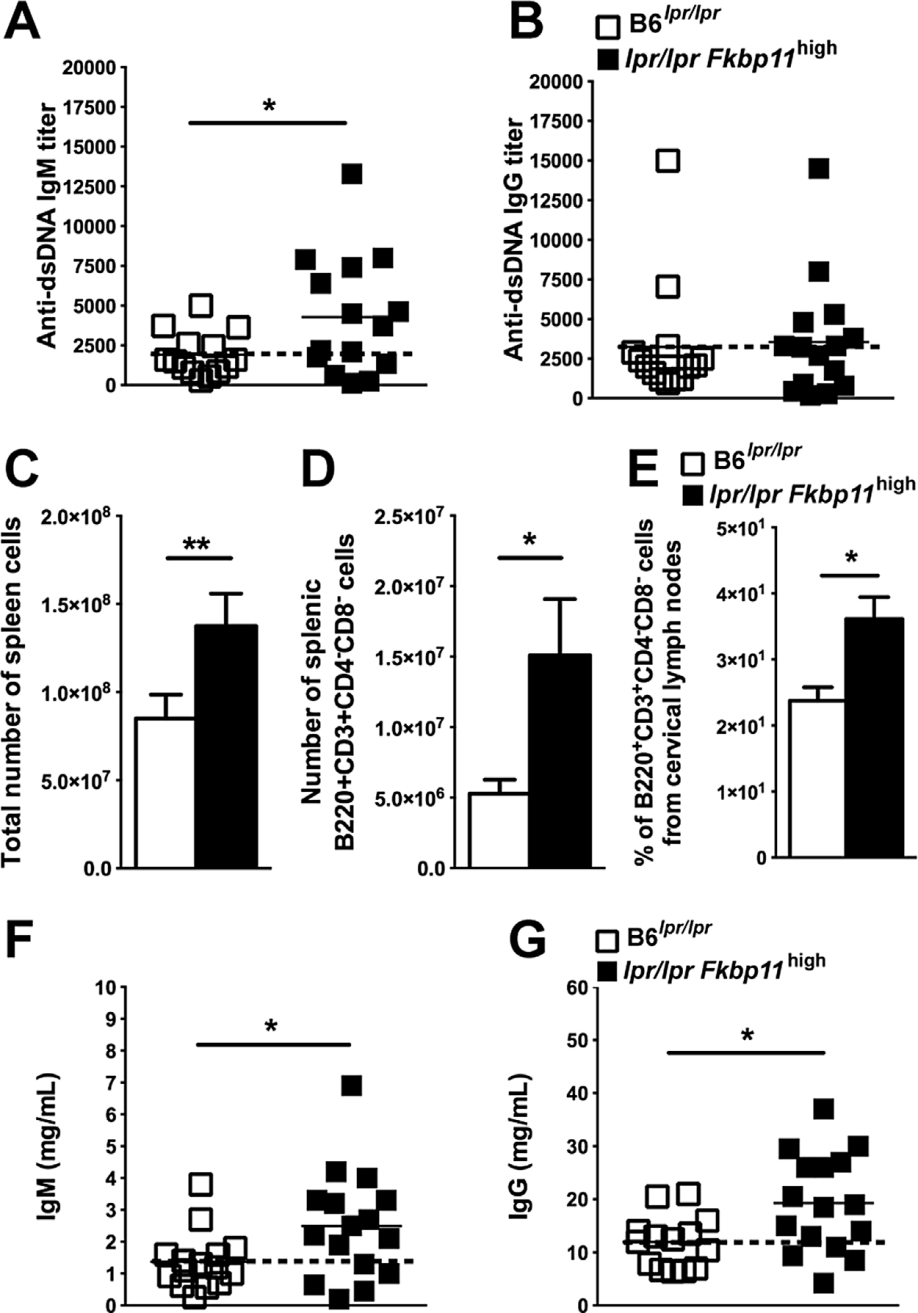
*Fkbp11* overexpression increases lymphoproliferation and hypergammaglobulinemia in *lpr/lpr* *Fkbp11*^high^ mice. (A, B) Sera from 9-month-old *lpr/lpr* *Fkbp11*^high^ and B6*^lpr/lpr^* littermate control mice were collected then titers of anti-dsDNA IgM (A) and IgG (B) were determined by ELISA. Each point represents the result for one animal. (C to E) Quantification of total number of splenic cells (C), DN (B220^+^CD3^+^CD4^-^CD8^-^) splenic cells (D), and of the proportion of DN lymph node cells (E) from 9-month-old B6*^lpr/lpr^* and *lpr/lpr Fkbp11*^high^ mice (A, B: n = 15 mice in each group; C: n = 9 mice in each group). (F, G) Sera from 9-month-old *lpr/lpr* *Fkbp11*^high^ and B6*^lpr/lpr^* mice were collected then total IgM (F) and IgG (G) titers were determined by ELISA. Each point represents the result for one animal. (*P < 0.05, **P < 0.01, Mann & Whitney test). (Error bars, SEM). The dotted lines indicate the means for control groups.

Considering the breakdown of anti-DNA B cell tolerance on C57BL/6 background, it was interesting to test the effect of *Fkbp11* overexpression added to a well-described autoimmune prone genetic defect. The B6*^lpr/lpr^* mice are characterized by the late appearance of anti-DNA antibodies related to the apoptosis defect linked to *Fas* deficiency 22. Thus, we crossed the *Fkbp11* mice on the *lpr/lpr* mice on C57BL/6 (B6*^lpr/lpr^*) background. High expression of *Fkbp11* on the B6*^lpr/lpr^* background did significantly increase anti-dsDNA IgM, but not anti-dsDNA IgG production (Fig. 5, A and B). The splenomegaly was increased when *Fkbp11* was overexpressed (Fig. 5C). In addition, the number of double negative (DN) T cells in spleen and lymph nodes were increased in *Fkbp11* mice on the B6*^lpr/lpr^* background (Fig. 5, D and E). Finally, the IgM and IgG hypergammaglobulinemia was amplified in B6*^lpr/lpr^* mice by the addition of *Fkbp11* overexpression (Fig. 5, F and G). Overall, these data showed that some traits of the immunopathology of B6*^lpr/lpr^* mice were exacerbated by *Fkbp11* overexpression.

### *Fkbp11* is implicated in the plasma cell differentiation program, upstream of Pax5

**Figure 6:**
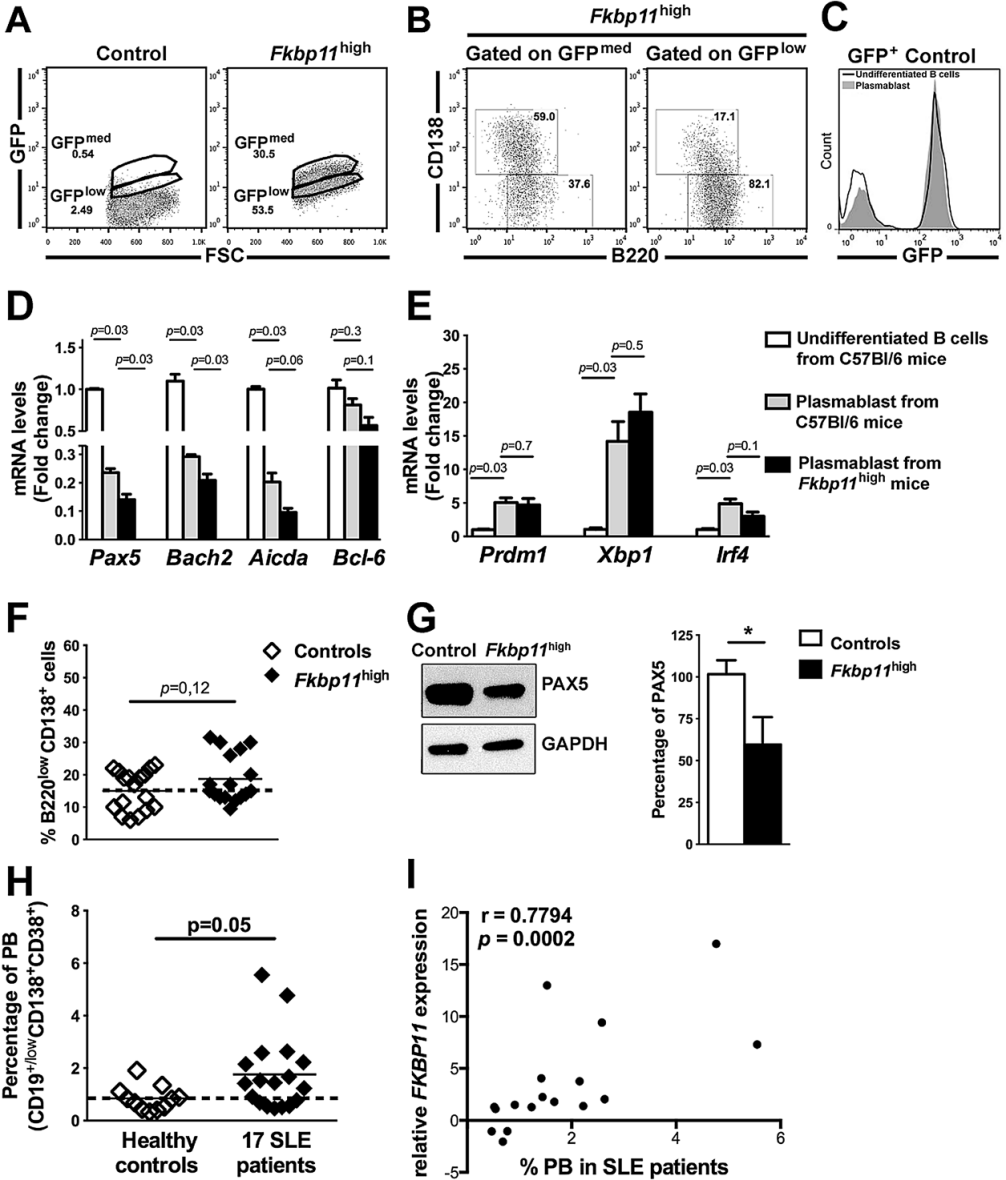
*Fkbp11* overexpression increases the initiation of plasma cell differentiation, and *FKBP11* overexpression in human SLE B cells is linked to plasma cell differentiation. (A–C) Splenic B cells were isolated from *Fkbp11*^high^ and littermate control mice and stimulated with LPS for 4 days *in vitro*, then analysed by flow cytometry. (A) Flow cytometry FSC-GFP diagram of B cells from control and *Fkbp11*^high^ mice. Numbers indicate the percentages of cells in each gate which are representative of n = 17 mice in each group. (B) Flow cytometry diagrams of PB (B220^+^CD138^low^) and undifferentiated B cells (B220^+^CD138^-^) in GFP^med^ and GFP^low^ populations from *Fkbp11*^high^ mice after 4 days of culture of isolated B cells. Numbers indicate the percentages of cells in each quadrant for n = 11 mice in each group. For PB and undifferentiated B cells, the difference between percentages in GFP^med^ and GFP^low^ populations were statistically significant (*P *< 0.0001, Mann & Whitney test). (C) GFP expression in undifferentiated B cells (black line) and PB (grey histogram) after 4 days of stimulation of isolated mature B cells from GFP^+^ control mice with LPS *in vitro*. (D to G) After 4 days of stimulation of purified splenic mature B cells from littermate control mice and *Fkbp11*^high^ mice with LPS for 4 days *in vitro*, the PB and undifferentiated B cells were sorted, then (D, E) the expression of master genes involved in PC differentiation was quantified by quantitative real time RT-PCR. Each sample was normalized to the endogenous control *Hprt1*. Each bar represents the level of mRNAs in sorted PB cells relative to undifferentiated B cells from control mice. (n = 4 littermate control mice and n = 4 *Fkbp11*^high^ mice; *P < 0.05, Mann & Whitney test; error bars, SEM), (F) the PB's percentages were quantified by flow cytometry. Each point represents the result for one animal. The result of Mann & Whitney test is indicated. The dotted lines indicate the means for control groups. (G) PAX5 protein expression from B cells stimulated with LPS during 4 days *in vitro* was analyzed by Western blot (n = 6 mice in each group). The quantification of PAX5's signal was done with ImageJ Software. (*P < 0.05, Mann & Whitney test) (Error bars, SEM). (H) Percentage of plasmablasts (PB) (CD19^+^CD138^+^CD38^+^) in healthy controls and in the 17 SLE quiescent patients. (I) Correlation between the percentages of PB in the 17 SLE quiescent patients and the *FKBP11* levels of expression (Spearman correlation test), for one of the two probe sets in our transcriptomic analysis (similar results were obtained with the second probe set: r = 0.7892, *P *= 0.0002, Spearman correlation test). The dotted lines indicate the means for control groups.

Considering that *FKBP11* expression is increased during plasma cell (PC) differentiation 23,24, and that anti-DNA production is increased in *Fkbp11* overexpressing mice on C57BL/6 and 56R backgrounds, we decided to study the consequences of *Fkbp11* overexpression on B cell differentiation. Known *in vitro* models of B cell differentiation, and LPS stimulation in particular, result in antibody secreting cells that are mostly B220^low^/CD138^+^ plasmablasts (PB), and consistently overexpress *Prdm1* (coding for Blimp1 protein) 25,26. Taking advantage of our transgenic construct which directly links GFP and *Fkbp11* expression, we followed the fate of GFP-expressing cells after LPS-induced *in vitro* PB differentiation. As shown in Figure 6A, at day 4, we observed in culture from *Fkbp11*^high^ mice a GFP^med^ and a GFP^low^ populations that are almost absent in culture from control mice. In addition, the GFP^med^ population contained the majority of PB, compared to the GFP^low^ population (51% of PB in GFP^med^ B cells, versus 10% in GFP^low^ B cells, *P *< 0.0001, Mann Whitney test) (Fig. 6B). We have shown by quantitative real-time RT-PCR that *Fkbp11* is most importantly (about 5 times, data not shown) overexpressed in sorted GFP^med^ B cells from *Fkbp11*^high^ mice, compared to sorted GFP^low^ B cells from the same mice, as expected with the T2A system which permits an equimolar expression of the two proteins. Therefore, it indicates that *Fkbp11* overexpression favored PB differentiation. In parallel, using splenic B cells from GFP^+^ control mice, we confirmed that this increase of GFP expression was not a consequence of ubiquitin promoter activation during LPS induced PB differentiation: indeed, GFP expression was not different in B220^+^CD138^−^ undifferentiated B cells and PB after 4 days of stimulation of isolated mature B cells with LPS *in vitro* (Fig. 6C). To better understand the role of *Fkbp11* overexpression on B cell differentiation, we quantified the expression of PC differentiation master genes 26–30 by quantitative real-time RT-PCR, in sorted PB and undifferentiated B cells from the transgenic *Fkbp11*^high^ mice and littermate control mice, after 4 days of culture of isolated mature B cells with LPS *in vitro*. As expected, in control mice, the expression of *Pax5*, *Bach2*, and *Aicda* is significantly decreased in sorted PB versus undifferentiated B cells (Fig. 6D), while the expression of *Prdm1*, *Xbp1*, and *Irf4* is increased (Fig. 6E). Interestingly, at the same time point, sorted PB from *Fkbp11*^high^ stimulated B cells expressed significantly lower *Pax5*, *Bach2*, and *Aicda* (Fig. 6D) (which are linked to the PC initiation program), than PB from littermate controls stimulated B cells. However, genes implicated in later stages of PB differentiation (*Prdm1* and *Xbp1*) were not significantly different in PB from *Fkbp11*^high^ stimulated B cells compared to PB from littermate controls stimulated B cells (Fig. 6E), and the percentages of PB were not significantly increased after 4 days of culture of B cells from *Fkbp11*^high^ mice, compared to control mice (Fig. 6F). The higher downregulation of *Pax5* expression in PB from *Fkbp11*^high^ stimulated B cells was confirmed at the protein level by Western-Blot analysis (Fig. 6G). In conclusion, considering the PC master gene differentiation program, *Fkbp11* overexpression initiates PC differentiation, acting upstream of Pax5 through a higher depression precisely of genes whose downregulation is implicated in the initiation of PC differentiation. The levels of transcription of lately expressed genes, like *Xbp1* and *Prdm1*, are not affected during our *in vitro* conditions perhaps because *Pax5* level is not the unique regulator of *Xbp1* expression. For example, the levels of expression of *Bcl6* and *Irf4*, which are also regulators of *Prdm1* (and consequently of *Xbp1*), are not different in PB from the two groups of mice (Fig. 6, D and E). In addition, *Xbp1* expression also appears to be influenced by a post-transcriptional mechanism involving the Unfolded Protein Response 31. Altogether, these results are consistent with the *in vivo* analysis of splenic B220^−^ CD138^+^ plasma cell population, which was not significantly increased in *Fkbp11*^high^ mice, compared to control mice (0.9 ± 0.6% for *Fkbp11*^high^ mice versus 1 ± 0.5% for littermate control mice).

Finally, to confirm the role of *Fkbp11* on the early phase of B cell terminal differentiation, we performed *Fkbp11* knock-down experiments in B cells from C57BL/6 mice, using a siRNA that targeting the murine *Fkbp11* gene (Supplementary Fig. S5A). This led to the opposite phenotype: expression of *Pax5*, *Bach2*, and *Aicda* was increased after culture of siRNA treated-B cells with LPS *in vitro*, compared to B cells treated with a control siRNA (Supplementary Fig. S5B). Taken together, these results confirmed a positive role of *Fkbp11* expression in the initiation of PC differentiation, and this effect is B cell intrinsic.

### *FKBP11* overexpression in human SLE B cells is linked to plasma cell differentiation

Human peripheral B cells contain a small fraction of PB (CD19^low/+^CD138^+^CD38^+^). We found a increase of the percentages of circulating PB in the 17 inactive SLE patients (containing the initial subgroup of five patients), compared to controls (1.76% in SLE patients versus 0.84% in controls, Fig. 6H). This minor increase cannot by itself explain the observed variation of *FKBP11* expression in total B cells during our transcriptomic analysis (ranging from −2 to 17, with two separate probe sets) 10. Furthermore, we found a correlation between the percentages of circulating PB and the *FKBP11* levels of expression (Spearman correlation test, *P* = 0.0002; Fig. 6I), suggesting that this overexpression favored PB differentiation, thus reinforcing the results of our *in vitro* studies with transgenic B cells. Taken together, these results suggest that *FKBP11* overexpression initiates PC differentiation, but other unknown molecular events were present in SLE patients to allow terminal PC differentiation.

## Discussion

*FKBP11* was identified by transcriptome analysis of human SLE B cells during clinically inactive disease. The higher expression of *FKBP11* mRNAs in these cells, compared to control B cells, led us to study the effects of *FKBP11* overexpression on B cell phenotype and on the development of autoimmunity.

For this purpose, we developed and validated a new model of ubiquitous *Fkbp11* overexpression in mice, using a lentigenic transgenesis system. These mice are named *Fkbp11*^high^, and show a 12 times overexpression of *Fkbp11* in purified splenic mature B cells.

The phenotype of *Fkbp11*^high^ mice included hyperplasia of lymphoid organs (with a more significant increase of B-cell subpopulations), an increase of basal IgG3 production, associated with an increase of B cell responsivity to a T-independent antigen *in vivo*. This phenotype was not correlated with an increase in basal B cell activation or after stimulation *in vitro*.

*Fkbp11* overexpression was sufficient to break B cell tolerance. This was characterized by a statistically significant increase of IgG anti-dsDNA production, with a sub-group of animals also secreting higher levels of IgM autoantibodies that targeted several autoantigens (including non-nuclear ones). Furthermore, the overexpression of *Fkbp11* exacerbated some features of autoimmunity in the B6*^lpr/lpr^* model. Finally, we used the well-documented 56R anti-DNA model on C57BL/6 background to study in more detail the mechanism of tolerance breakdown when *Fkbp11* is overexpressed. The 56R model consists in a high affinity heavy chain that binds to ssDNA and a dsDNA and is knocked into the heavy chain locus by gene targeting 16,17. Initial studies of B cell tolerance in the 3H9 and 56R anti-DNA KI model were conducted on BALB/c background, and have shown that tolerance to nuclear antigens was maintained through a variety of mechanisms, including deletion and receptor editing for high-affinity anti-dsDNA B cells, and anergy of anti-ssDNA B cells, that block autoantibody secretion. The 56R model on C57BL/6 background was shown to be more susceptible to a loss of tolerance 16,32. Indeed, Liu and colleagues have crossed 3H9 and 56R models (on C57BL/6 background) to the lupus susceptible Sle2z congenic mice and have shown that 56R model was more prone to B cell tolerance breakdown, which was characterized by blockade of receptor editing, a skewing toward marginal zone B cells and preplasmablats, and an increased secretion of autoantibodies 16. Our results on 56R/*Fkbp11*^high^ mice demonstrate that the production of IgM anti-ssDNA autoantibodies (corresponding to the highest proportion of autoantibodies produced by C57BL/6-56R mice) is increased. This phenotype was not readily explained by detectable abnormalities in central autoreactive B cell deletion, autoreactive B cell anergy nor autoreactive B cell receptor editing. Thus, if *Fkbp11* overexpression by its own was indeed sufficient to induce a breakdown of B cell tolerance, which is a remarkable feature of SLE patients 33, further experiments are needed to explain the precise mechanisms. In addition, we cannot exclude a role of *Fkbp11* overexpression in other cell types than B cells in the observed effects.

The increased number of circulating PC is a feature of many patients during SLE, even during inactive phases of the disease 34,35. Since *FKBP11* is overexpressed during PC differentiation 23,24, we asked about the consequences of *Fkbp11* overexpression on B cell differentiation when *Fkbp11* is overexpressed at a B-cell maturation stage (plasmablast, PB) preceding PC differentiation. It is currently accepted that Pax5 is one of the master proteins controlling PC differentiation 25,26. However, little is known about the factors that control Pax5 expression 28. Our data showed that, after 4 days of stimulation of purified splenic mature B cells with LPS *in vitro*, pro-germinal center B cell genes i.e., *Pax5*, *Bach2*, and *Aicda* (the last two being positively controlled by Pax5) exhibit reduced expression in PB from *Fkbp11*^high^ mice, as compared to PB from control mice, and this effect is B cell intrinsic. However, this effect of *Fkbp11* overexpression on the PC differentiation genetic program is incomplete since the expression of pro-plasmacytic genes (*Prdm1*, *Xbp1*) was not intensified. Thus, the expression of *Fkbp11* is not only increased during PC maturation, suggesting a potential role of *Fkbp11* in this process, but the overexpression of this gene at a B-cell maturation stage preceding PC differentiation, can promote the initiation of this process. According to our *in vitro* results, *Fkbp11* overexpression is not sufficient to induce a complete PC differentiation genetic program indicating that full PC differentiation requires additional signals that are not present in our *in vitro* PC differentiation system, but could be present in lupus patients. Importantly, our results localize FKBP19 protein (encoded by *Fkbp11* gene) upstream of Pax5 (by direct or indirect effect) during the PC differentiation pathway. FKBP19, as the other members of FKBP family, seems to have a double function with a PPIase activity and a putative chaperone activity 36. The PPIase activity catalyzes a cis/trans peptidyl-prolyl isomerization of target proteins, which could have an effect on the interactions between two particular partners and on their activation. It is also known that PPIase enzymes interact with a diverse range of intracellular and extracellular targets, however the molecular interactions between PPIase and target proteins are weak. It is tempting to inhibit FKBP19 PPIase activity in order to evaluate its precise role in the development of this phenotype *in vivo*. However, today there isn't any FKBP19-specific inhibitor available 37. FK506 inhibits the PPIase activity of some FKBPs, particularly FKBP13, therefore it is not specific to FKBP19, and binds FKBP19 200 times less than FKBP13 12. Other FKBP's inhibitors exist. They are FK506 structural analogs, but they also lack specificity for one particular member of the family 38. FKBPs, and potentially FKBP19, are also known to have a chaperone activity, which seems to be independent of the PPIase activity 36. FKBP19 could therefore play a chaperone role for proteins implicated in the regulation of Pax5, for example, or other targets that remain to be defined in future experiments.

Considering the complexity of SLE immunopathology, our results yield novel insights into the pathogenesis of the disease because they show that overexpression of a single gene, *FKBP11,* in B cells, is sufficient to induce some biological characteristics of SLE. The dissection of molecular pathways targeted by the FKBP19 protein, and the analysis of *FKBP11* expression in larger cohorts of patients, looking for correlation between *FKBP11* levels in B cells and clinical phenotypes of SLE patients, may lead to the identification of new molecular targets in SLE.

## Materials and Methods

### Patients

Patients with SLE fulfilling at least 4 diagnostic criteria according to the American College of Rheumatology 39 were prospectively included provided that they were in a quiescent phase of the disease (SLEDAI score less than 4) and received minimal treatment [no immunosuppressive drugs; if they needed steroids, the patients were not treated with more than 10 mg of prednisone per day (4 patients)]. 10 patients were treated with hydroxychloroquine 10. Purified B cells from 17 patients (and 9 age- and sex-matched controls) were subjected to a pangenomic transcriptome analysis (Affymetrix GeneChip human genome U133 plus 2.0). This study was approved by the ethics committee of the Hôpitaux Universitaires de Strasbourg and patients gave their written informed consent.

### DNA constructs

The self-inactivating lentiviral vector pTRIP-Ubi-GFP-T2A-*Fkbp11*-ΔU3 (Supplementary Fig. S1A) used to produce transgenic mice was constructed as follows: the lentiviral pTRIP-Ubi-GFP-ΔU3 (used for the production of GFP^+^ lentigenic control mice) was obtained by substituting the EF1α promoter for the human Ubiquitin C (UbiC) promoter from pUbi-JunB (generously provided by P. Angel 14) in MluI and BamHI sites of pTRIP-EF1α-GFP-ΔU3 (kindly provided by P. Charneau 40). An insert containing BamHI restriction site, GFP sequence without STOP codon, a multiple cloning site (MCS) and XhoI sequence was produced by PCR, and then used to replace GFP sequence in pTRIP-Ubi-GFP-ΔU3, in order to produce pTRIP-Ubi woSTOP-MCS. This MCS notably contains AscI then NdeI restriction sites. The CDS of murine *Fkbp11* mRNA (606 bp, NM024169) was amplified by PCR from total murine splenocytes cDNA with the following primers: Forward 5′-CACCATGACCCTGCGCCCCTCACT-3′, and Reverse 5′-TTTCTTTTTGCTCTTGTTTCGTTTCTCTTCCTTGAGC-3′(Sigma, St Louis, MO, USA). The PCR conditions were: 94°C for 5 min; 35 cycles at 94°C for 30 s, 54.4°C for 30 s and 72°C for 1 min. The PCR product was subcloned into the pCR2.1 TA cloning vector (Invitrogen, Carlsbad, CA, USA). Then AscI restriction site and the T2A sequence were added upstream *Fkbp11* CDS, and NdeI restriction site was added downstream *Fkbp11* CDS, by PCR amplification on *Fkbp11*-pCR2.1 TA cloning vector, using the following primers: Forward 5′-AAAGGCGCGCCTT*GAGGGCAGAGGAAGTCTTC TAACATGCGGTGACGTGGAGGAGAATCCCGGCCCT*ATGACCCTGAGCCCT-3′; Reverse: 5′-AAACATATGTTATTTCTTCTTACTCTTGTTT-3′. The PCR conditions were: 94°C for 5 min; 25 cycles at 94°C for 30 s, 58.4°C for 30 s and 72°C for 1 min. The final PCR product (672 bp) was cloned into the pCR2.1 TA cloning vector (Invitrogen) to obtain pCR2.1-*Fkbp11*-T2A vector. Finally, the *Fkbp11*-T2A insert was subcloned into pTRIP-Ubi woSTOP-MCS to obtain pTRIP-Ubi-GFP-T2A-*Fkbp11*-ΔU3 lentiviral vector.

### Virus production and titration

Lentiviral particles were produced by transient transfection of 293T cells as described 40.

**Mice**

In order to produce *Fkbp11* lentigenic mice, 10 pL of lentivirus at 5 × 10^8^ IU/ml was injected into the perivitelline space of one-cell stage C57BL/6 mouse embryos (SEAT, Villejuif, France). Embryos were reimplanted into pseudopregnant females. One single-copy *Fkbp11* founder (see following paragraph) was selected for further analysis. These *Fkbp11*^high^ transgenic mice were then maintained on C57BL/6 background. Progenies were screened by PCR on tail DNA, using following primers: Forward: 5′-CACCATGACCCTGCGCCCCTCACT-3′ and Reverse: 5′-CCAGGCACAATC AGCATTGGTAGCT-3′. The PCR conditions were: 94°C for 5 min; 30 cycles at 94°C for 30 s, 55°C for 30 s and 72°C for 40 s; size of PCR product: 680bp). *lpr/lpr Fkbp11*^high^ mice were generated by crossing C57BL/6*^lpr/lp^^r^* mice (B6.MRL-*Fas^lpr^*/J; The Jackson Laboratory) with *Fkbp11*^high^ mice and by intercrossing between the littermates. 56R mice on C57BL/6 background 16 were kindly provided by M. Weigert (Department of Pathology, University of Chicago, Chicago, IL, USA). 56R/*Fkbp11*^high^ mice were generated by crossing 56R mice with *Fkbp11*^high^ mice. In each experiment, littermate mice were used as controls. The experiments were conducted on both males and females, except for experiments on *lpr/lpr* *Fkbp11*^high^ mice and B6*^lpr/lpr^* littermate controls, which was conducted exclusively on females. All animal experiments were performed in an animal facility, which is authorized by the “Direction départementale des services vétérinaires” (Strasbourg, France) and protocols were approved by the “Comité d'éthique en matière d'Experimentation Animale de Strasbourg” (CREMEAS, approval number AL/02/15/09/11).

### Analysis of the number of proviral integrations by Southern blotting

The number of proviral integrations was analyzed by Southern blot (Supplementary Fig. S1C) on AvaII- and EcoNI-digested tail DNA, as previously described 40. The probe (1027 bp, (Supplementary Fig. S1B) used was the result of the PCR on pTRIP vector with the following primers: 5EcoNI (5′-CAGGGACTTGAAAGCGAAAG-3′) and 3EcoNI (5′- GCTTGTGTAATTGTTAA TTTCTCTGTC-3′).

### Cell preparation and culture

For activation of splenic mature B cells *in vitro,* spleen cells were plated at 1.10^6^ cells/ml in culture medium composed of RPMI-1640 (Lonza) containing 10% (v/v) FCS (PAN), 50 µM β-Mercaptoethanol (Gibco, Paisley, Scotland), 1% Penicillin/Streptomycin (Gibco), 10mM HEPES (Lonza, Verviers, Belgium), 1 mM Sodium Pyruvate (Lonza). Cells were stimulated with LPS alone (10 μg/ml; Sigma), F(ab')_2_ anti-mouse IgM (5 μg/ml; Jackson Immunoresearch), CG-50 (a large DNA fragment containing tandem repeats of hypomethylated CpG which is used to specifically stimulate anti-DNA B cell), or CG-Neg negative control (a large DNA fragment which is devoid of hypomethylated CpG) (0.2 ng/μl), for the indicated time. CG-50 and CG-Neg (HIV-CG^−^) plasmids were kindly provided by Prof. Marshak-Rothstein, and were produced as previously described 18,19. For plasmablast differentiation with LPS *in vitro*, splenic mature B cells (CD43^−^) were purified using B-cell isolation kit (anti-CD43 (Ly-48) microbeads) according to the supplier's protocol (Miltenyi Biotech, Auburn, CA, USA). B cells were then plated at 1.10^6^ cells/mL and were stimulated for 4 days with LPS (10 μg/ml, Sigma) using the same medium as for total splenic cells culture. For knock-down experiments, the efficiency of anti-*Fkbp11* siRNA (reference 102718384, Qiagen), compared to a control siRNA (reference 1027280, Qiagen) was tested in L929 murine fibroblasts, using HiPerFect transfection reagent (Qiagen, Hilden, Germany) according to Qiagen protocol specific for fibroblasts, at 24h and 48h after transfection with the siRNAs. For knock-down experiments in B cells, splenic mature B cells were sorted as explained above, and then were transfected with the anti-*Fkbp11* siRNA or the control siRNA, using HiPerFect transfection reagent (Qiagen), according to Qiagen protocol specific for suspension cell lines, before adding LPS in the culture medium.

### Quantitative real-time RT-PCR analysis

mRNA was prepared with RNeasy Kit (Qiagen) and cDNA was obtained with High Capacity Reverse Transcription Kit (Applied Biosystems, Foster City, CA, USA). Quantitative real-time PCR was performed on 10 ng cDNA using Taqman Universal Mastermix (Applied Biosystems) and Assays-on-Demand probes (*Hprt1*: Mm01318743_m1, *Fkbp11*: Mm00470261_m1, *Pax5*: Mm00435501_m1, *Blimp1*: Mm01187285_m1, total *Xbp1*: Mm00457357_m1, *Bach2*: Mm00464379_m1, *Aicda*: Mm00507771_m1). Each sample was amplified in triplicate in a StepOnePlus real-time PCR machine (Applied Biosystems). mRNA levels were calculated with the StepOne v2.1 software (Applied Biosystems), using the comparative cycle threshold method, and normalized to the endogenous control *Hprt1*.

### Analysis of RS rearrangements

Splenic follicular (Fo: CD23^+^CD21^low^), marginal zone (MZ: CD23^-^CD21^high^), and transitional 1 (T1: CD23^−^CD21^−^) IgM^a+^ B220^+^ B cells were sorted by flow cytometry. Then RS rearrangement levels were determined as described previously 20.

### Vκ light chain sequencing

Light chain high throughput sequencing was performed using a two-step PCR. In the first step, Vκ-Jκ1 and Vκ-Jκ5 rearrangements were amplified using cocktails of primers (Forward: Vκ FW1 5′-GAY ATT GTG MTS ACM CAR WCT MCA-3′; Reverse: Jκ1 5′-ACG TTT GAT TTC CAG CTT GG-3′, Jκ5 5′-ACG TTT CAG CTC CAG CTT G-3′). In the second step, band purified amplicons were re-amplified using primers that were modified to include illumina sequencing barcodes and adaptors, using protocols available on the illumina website (http://supportres.illumina.com). Equimolar amounts of each sample library were subsequently sequenced on the Illumina Mi-seq using 2 by 250 base paired end reads. Sequences extending from the Jκ end were analyzed using the IMGT high-throughput sequence analysis server and only unequivocally identified Vk genes were included in the analysis.

### Immunoblot analysis

Proteins were extracted by standard techniques. Primary antibodies and dilutions were as follows: Rabbit anti-PAX5, 1:1000 (Cell Signaling, #12709); Rabbit anti-FKBP19, 1:500 (Abcam, ab175570); Mouse anti-GFP, 1:1000 (Roche, 11814460001); Goat anti-Actin-HRP, 1:5000 (Santa Cruz, sc-1615); Rabbit anti-GAPDH-HRP, 1:2000 (Santa Cruz, sc-25778). Secondary antibodies and dilutions were as follows: sheep anti-mouse-HRP, 1:10 000 (GE Healthcare, NA3910V); Donkey anti-rabbit-HRP 1:10 000 (GE Healthcare, NA9340V).

### Flow cytometry analysis and cell sorting

Cell phenotype was performed on lymph nodes, splenic and bone marrow lymphoid populations by four-color fluorescence analysis according to standard protocols. The following antibodies and reagents were used: PerCP, PE, or APC anti–mouse B220, CD3, CD4, CD8, CD19, CD21, CD23, CD44, CD69, CD86, I-A/I-E, CD25, CD138, and IgM (all from BD Biosciences). Propidium iodide was used for live-dead discrimination. For proliferation analysis, cells were permeabilized after extracellular staining and fixed with the cytofix/cytoperm permeabilization kit (BD Biosciences), then stained with the PerCP Cy5.5 anti-Ki67 (BD Biosciences). Cells were analyzed using a FACSCalibur. We then analyzed data with FlowJo software (Treestar). Bone marrow and splenic B-cell subsets (Supplementary Fig. S4) on the one hand, and PB and undifferentiated B cells after 4 days of culture with LPS (Fig. 6) on the other hand, were sorted by flow cytometry using a FACS Aria cell sorter (BD Biosciences), after staining with the antibodies indicated in the legend of the figures.

### Antibody detection by ELISA

Total IgG, IgG1, IgG2b, IgG3 or IgM levels were measured in serum from 8-month-old mice, and in supernatants after 3 days of stimulation, as previously described 11. Anti-thyroglobulin, -dsDNA, -actin, and RF titers, as well as anti-OVA and anti-NP specific antibodies were measured as previously described 11.

### Immunization

Six- to eight-week-old mice were injected intraperitoneally at days 0, 10, and 20 with 100 µg NP-LPS (Biosearch Technologies). Serum was collected before each injection and at day 30. For immunization experiments with ovalbumin (OVA), six- to eight-week-old mice were injected intraperitoneally with 100 μg OVA (Sigma) in complete Freund's adjuvant (Sigma) at day 0, in incomplete Freund's adjuvant at day 10 and in PBS at day 20. Serum was collected before treatment, and at every time point before the injection.

### Statistical analysis

Statistical significance was calculated with a two-tailed Mann & Whitney test or unpaired t test with Welch's correction, Wilcoxon match-pairs test, or Spearman correlation test, using Prism software (GraphPad).

## Supporting Information

Figure S1 : Creation of *Fkbp11* and control GFP+ lentigenic mice, and expression of *Fkbp11* in GFP+ control mice. **Figure S2**: Basal activation of B and T cells is not modified in *Fkbp11*^high^ mice compared to control mice. **Figure S3**: Analysis of deletion and anergy in 56R/*Fkbp11*^high^ mice. **Figure S4**: *Fkbp11* expression in B cell subpopulations in wild-type mice.
