# Supplementary material for: Overexpression of Fkbp11, a feature of lupus B cells, leads to B cell tolerance breakdown and initiates plasma cell differentiation
Source: Immun Inflamm Dis. 2015 Jun 18;3(3):265–79. doi: 10.1002/iid3.65 (PMC4578525; doi:10.1002/iid3.65)
Supplement: Figure S1 — : Creation of Fkbp11 and control GFP+ lentigenic mice, and expression of Fkbp11 in GFP+ control mice. Figure S2: Basal activation of B and T cells is not modified in Fkbp11high mice compared to control mice. Figure S3: Analysis of deletion and anergy in 56R/Fkbp11high mice. Figure S4: Fkbp11 expression in B cell subpopulations in wild-type mice. [file iid30003-0265-sd1.doc]

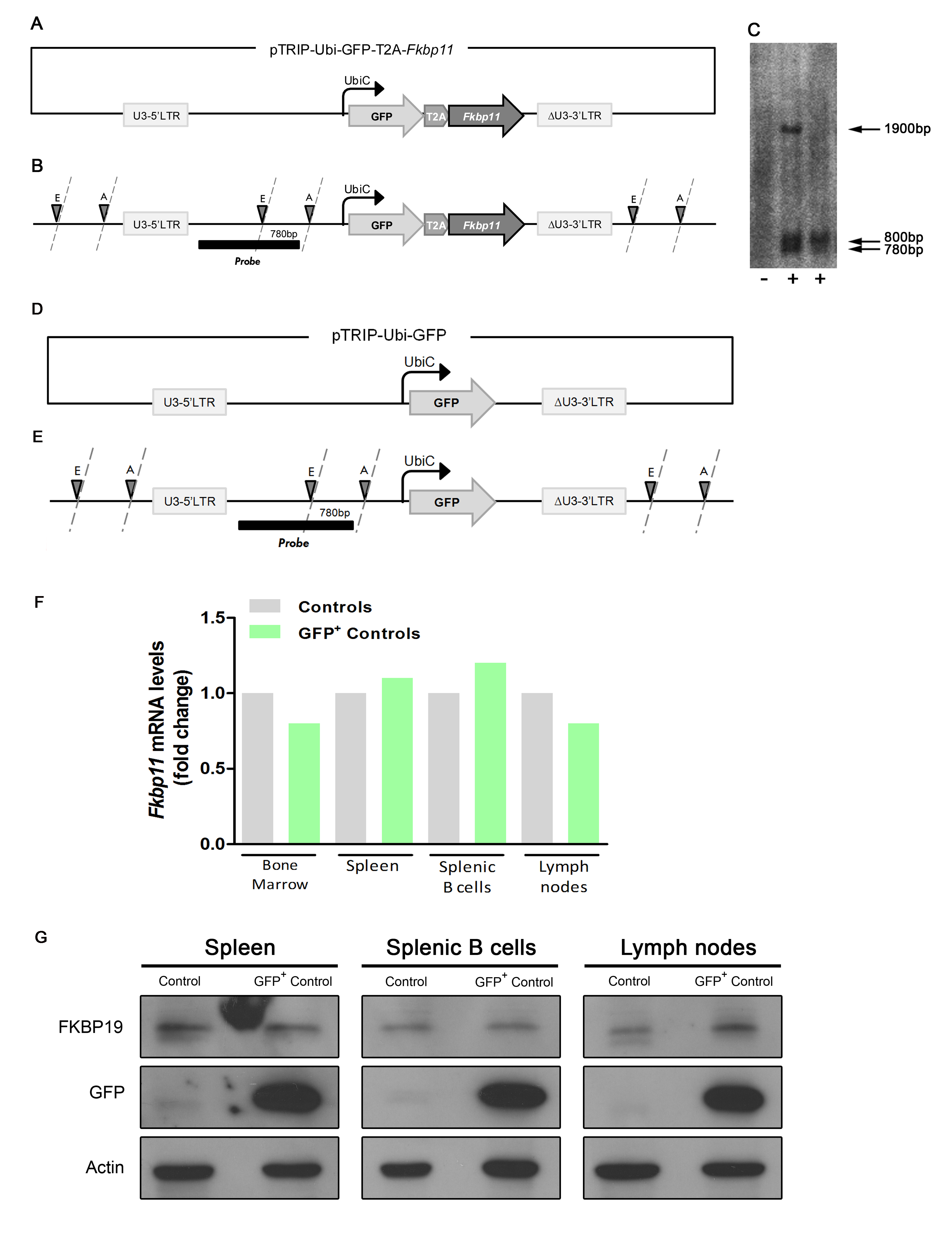


**Supplementary Fig. 1.** **Creation of** ***Fkbp11* and control GFP+ lentigenic mice, and expression of *Fkbp11* in GFP+ control mice.**

(**A**) Schematic representation of the pTRIP-Ubi-GFP-T2A-*Fkbp11* lentiviral vector. Restriction sites: E: EcoNI; A: AvaII. UbiC: human Ubiquitin C promoter. (**B**) Schematic representation of pTRIP-Ubi-GFP-T2A-*Fkbp11* provirus integrated in the genome of *Fkbp11* transgenic mouse line. (**C**) Southern blot analysis of tail DNA from littermate control mice (-) and lentigenic mice (+). EcoNI–AvaII–digested DNA from pTRIP-*Fkbp11*-positive mice (+: second and third lanes) and pTRIP-*Fkbp11*-negative control littermate mouse (-: first lane) was hybridized with the 5′ flanking probe, as indicated in **B**. Each integration will be visualized with the 780 bp band, which is common to all integrations, plus one additional band with superior size, in the case of an unique provirus integration (third lane: example for the *Fkbp11*^high^ line), or two additional bands in the case of two integrations (second lane: example for another mouse with two integrations). (**D**) Schematic representation of the pTRIP-Ubi-GFP lentiviral vector. Restriction sites: E: EcoNI; A: AvaII. UbiC: human Ubiquitin C promoter. (**E**) Schematic representation of pTRIP-Ubi-GFP provirus integrated in the genome of the control GFP^+^ lentigenic mouse line selected for this study. (**F**) Quantitative real time RT-PCR analysis of *Fkbp11* mRNA expression in total bone marrow cells, splenocytes, purified splenic mature B cells and total lymph node cells from GFP^+^ control mice and littermate control mice. Each sample was normalized to the endogenous control *Hprt1*. Each bar represents the level of *Fkbp11* mRNA in GFP^+^ mice relative to littermate control mice. (**G**) Western-Blot analysis of FKBP19 and GFP expression in splenocytes, purified splenic mature B cells and total lymph node cells from GFP^+^ control mice and littermate control mice. Actin was used as loading control.


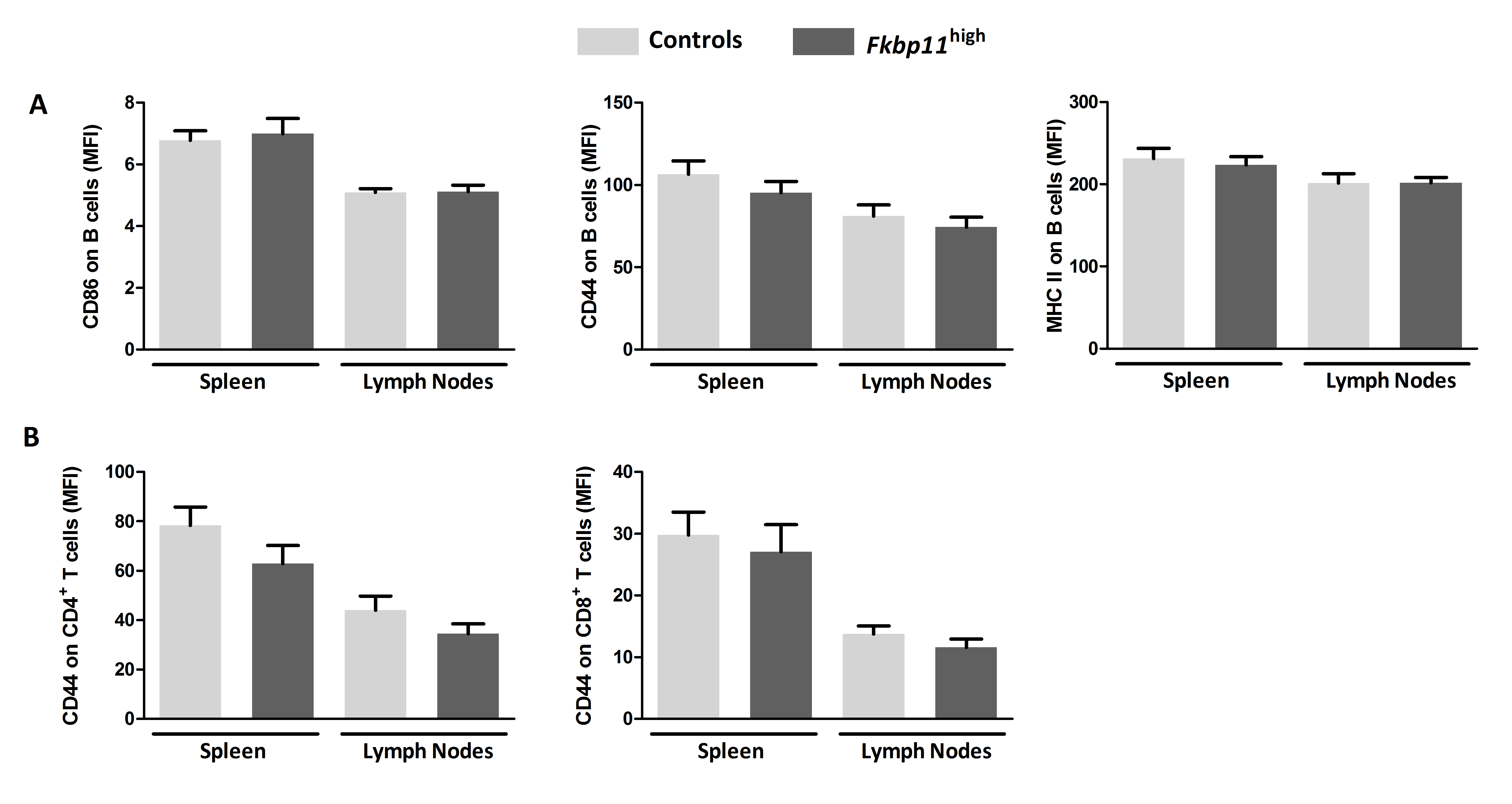


**Supplementary Fig. 2. Basal activation of B and T cells is not modified in *Fkbp11*^high^ mice compared to control mice.**

Flow cytometry analysis of cell surface expression (Mean Fluorescence Intensity, MFI) of CD86, CD44 and CMH II activation markers on splenic B cells (A) and CD44 on CD4^+^ and CD8^+^ splenic T cells (B) from 8 month-old *Fkbp11*^high^ and littermate control mice (controls) (*n*=14 for *Fkbp11*^high^, *n*=15 for control mice). (Error bars, SEM).


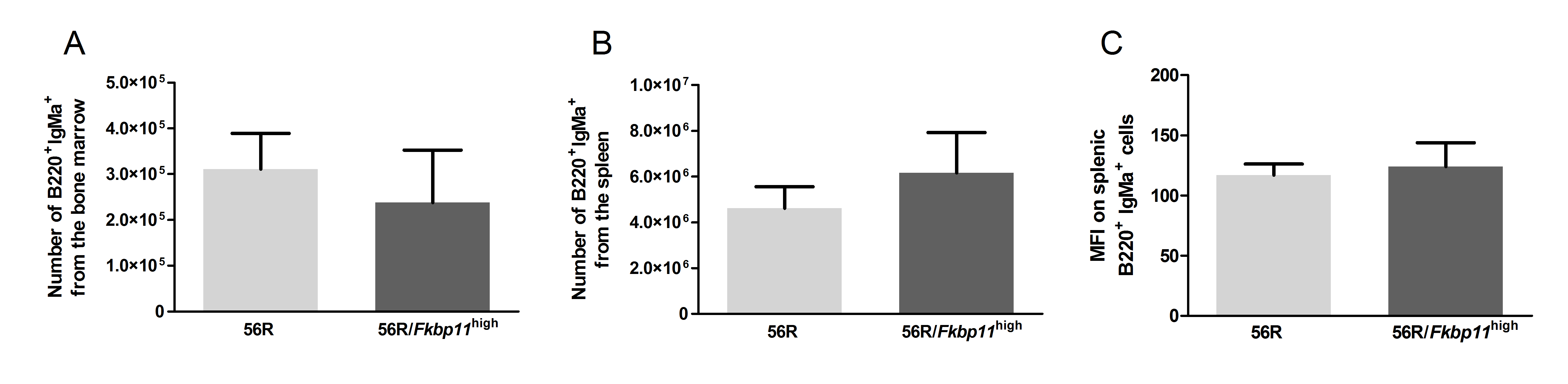


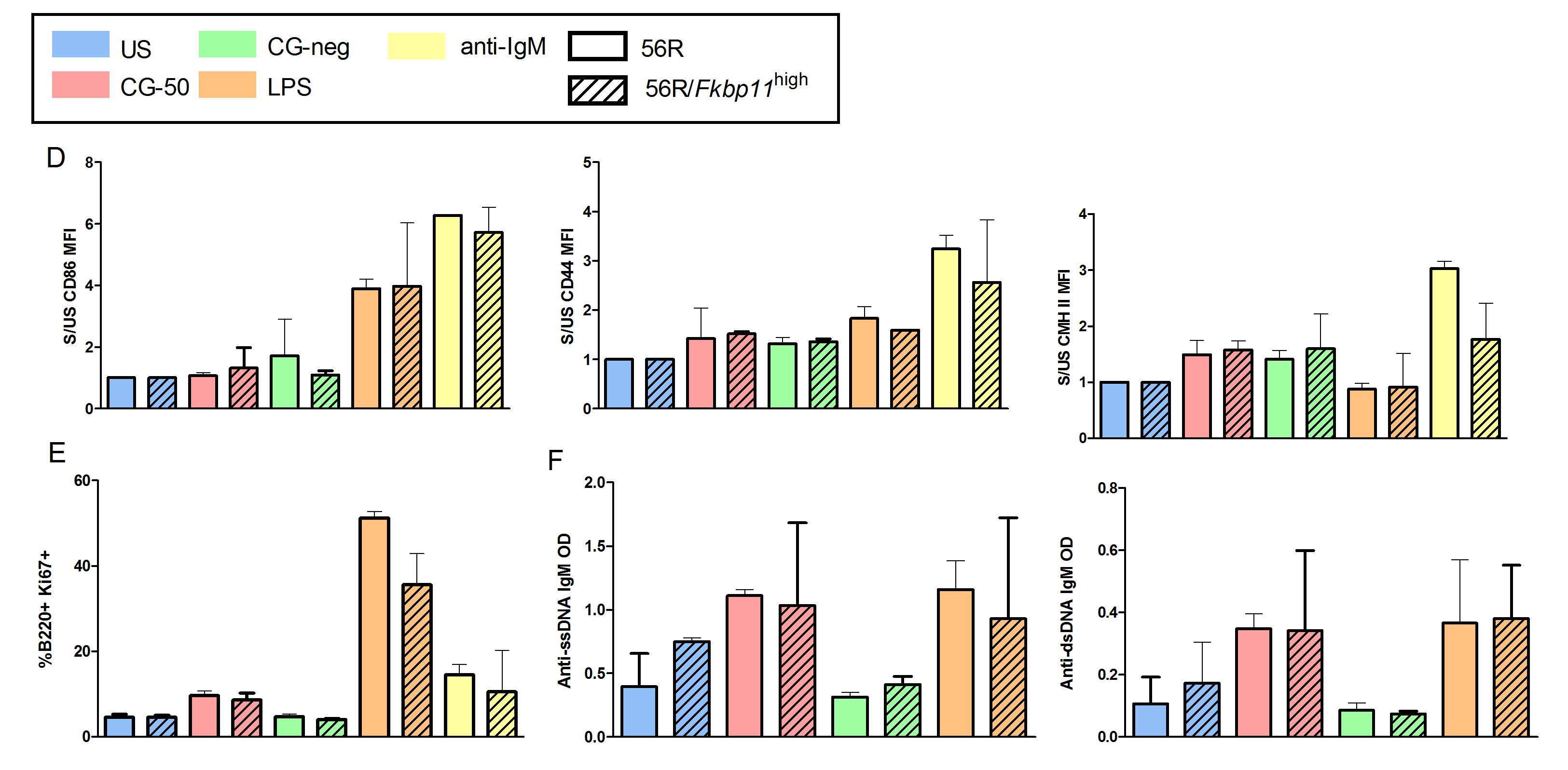


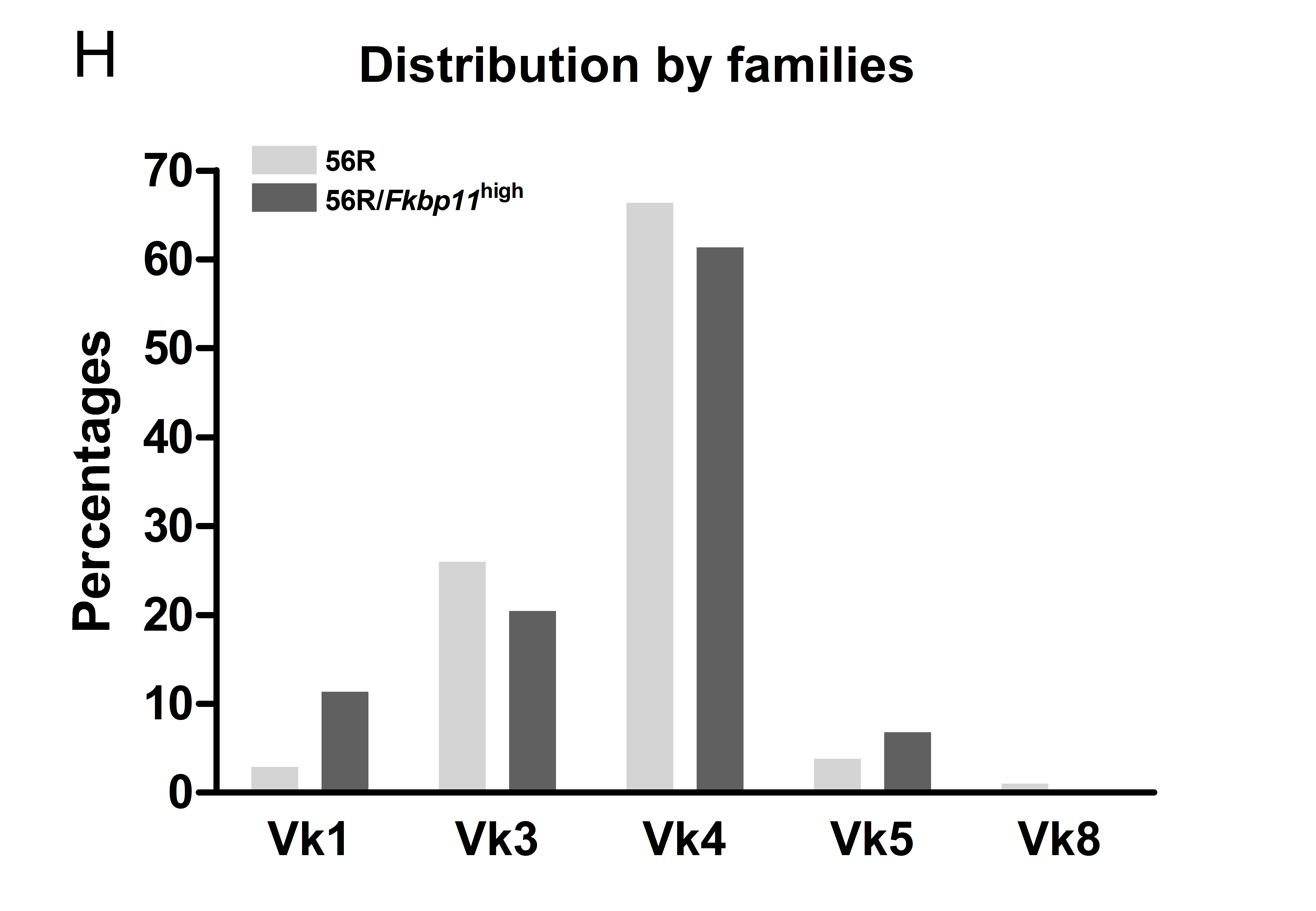

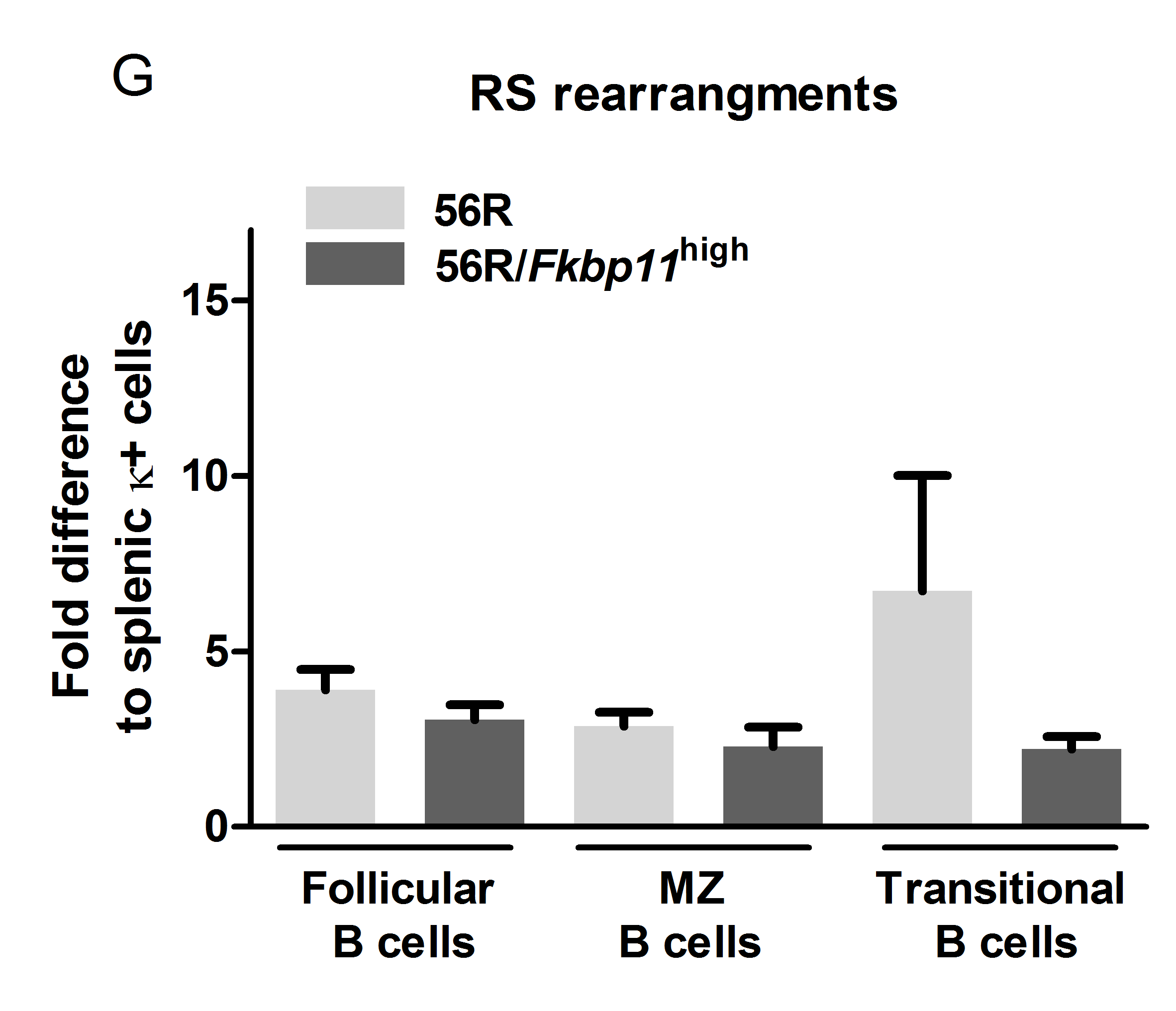


**Supplementary Fig. 3. Analysis of deletion and anergy in 56R/*Fkbp11*^high^ mice**.

(**A, B**). Quantification of bone marrow (**A**) and splenic (**B**) IgM^a+^ B cells from 56R/*Fkbp11*^high^ (*n=*5*)* and 56R littermate (*n=*5*)* 12-month-old mice after flow cytometry analysis. (**C**) Analysis of IgM^a^ Mean Fluorescence Intensity (MFI) on splenic B cells from 56R/*Fkbp11*^high^ (*n=*5*)* and 56R littermate (*n=*5*)* 12‑month-old mice*.* (**D**) Flow cytometry analysis of activation markers on splenic B cells from 56R/*Fkbp11*^high^ and 56R littermate 4-month-old mice (*n=*2), after stimulation with different stimuli (CG-50, a large DNA fragment containing tandem repeats of hypomethylated CpG which is used to specifically stimulate anti-DNA B cells, CG-Neg, a large DNA fragment which is devoid of hypomethylated CpG and is used as a negative control, LPS, or anti-IgM) for 3 days *in vitro*. Ratio of MFI on stimulated (S) cells versus unstimulated (US) cells are represented for CD86, CD44 and MHC II. (**E**) Cell proliferation was analyzed by flow cytometry by the percentage of B220^+^Ki67^+^ cells after 3 days of culture. (**F**) The production of anti-ssDNA (left) and anti-dsDNA IgM (right) in culture supernatants was quantified by ELISA (OD: optical densities). (Error bars, SEM). **(G)** Splenic T1 (IgM^a+^CD23^-^CD21^-^), marginal zone (IgM^a+^CD23^-^CD21^high^) and follicular (IgM^a+^CD23^+^CD21^low^) B220^+^ B cells were sorted from 56R/*Fkbp11*^high^ (*n=*4) and 56R littermate mice (*n=*4), then Vκ-RS rearrangement levels were quantified by quantitative PCR. Data are presented as the fold difference relative to the mean RS level in C57BL/6 splenic B220^+^IgM^+^k^+^ B cells (Error bars, SEM). (**H**) Distribution (percentages) of Vκ sequences in sorted T1 cells from 56R/*Fkbp11*^high^ (*n=*4) and 56R littermate mice (*n=*4).


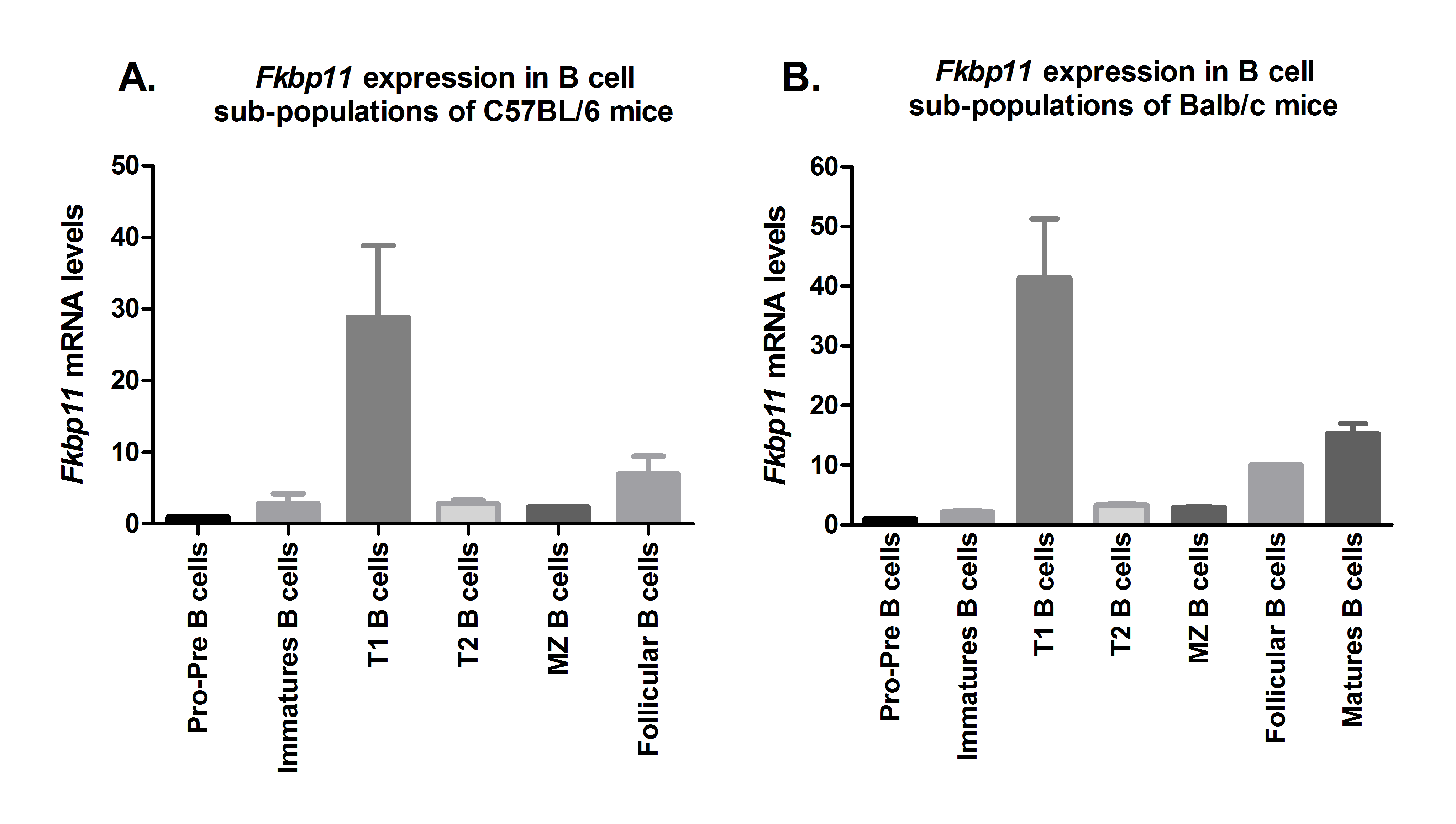


**Supplementary Fig. 4. *Fkbp11* expression in B cell subpopulations in wild-type mice.**

(**A**) Quantitative real time RT-PCR analysis of *F**kbp11* mRNA expression in FACS-sorted B cell subsets from C57BL/6 (*n*=2) (**A**) and Balb/c (*n*=2) (**B**) 4-month-old mice: Bone marrow Pro/PreB (B220^+^IgM^-^), Immature (B220^med^IgM^+^) and Mature recirculating (B220^high^IgM^+^, only in **B**); Splenic T1 (IgM^+^CD23^-^CD21^-^), T2 (IgM^+^CD23^+^CD21^high^), marginal zone (IgM^+^CD23^-^CD21^high^) and follicular (IgM^+^CD23^+^CD21^low^). Each sample was normalized to the endogenous control *Hprt1*. Each bar represents the level of *Fkbp11* mRNA relative to Pro-PreB subpopulation. (Error bars, SEM).

**
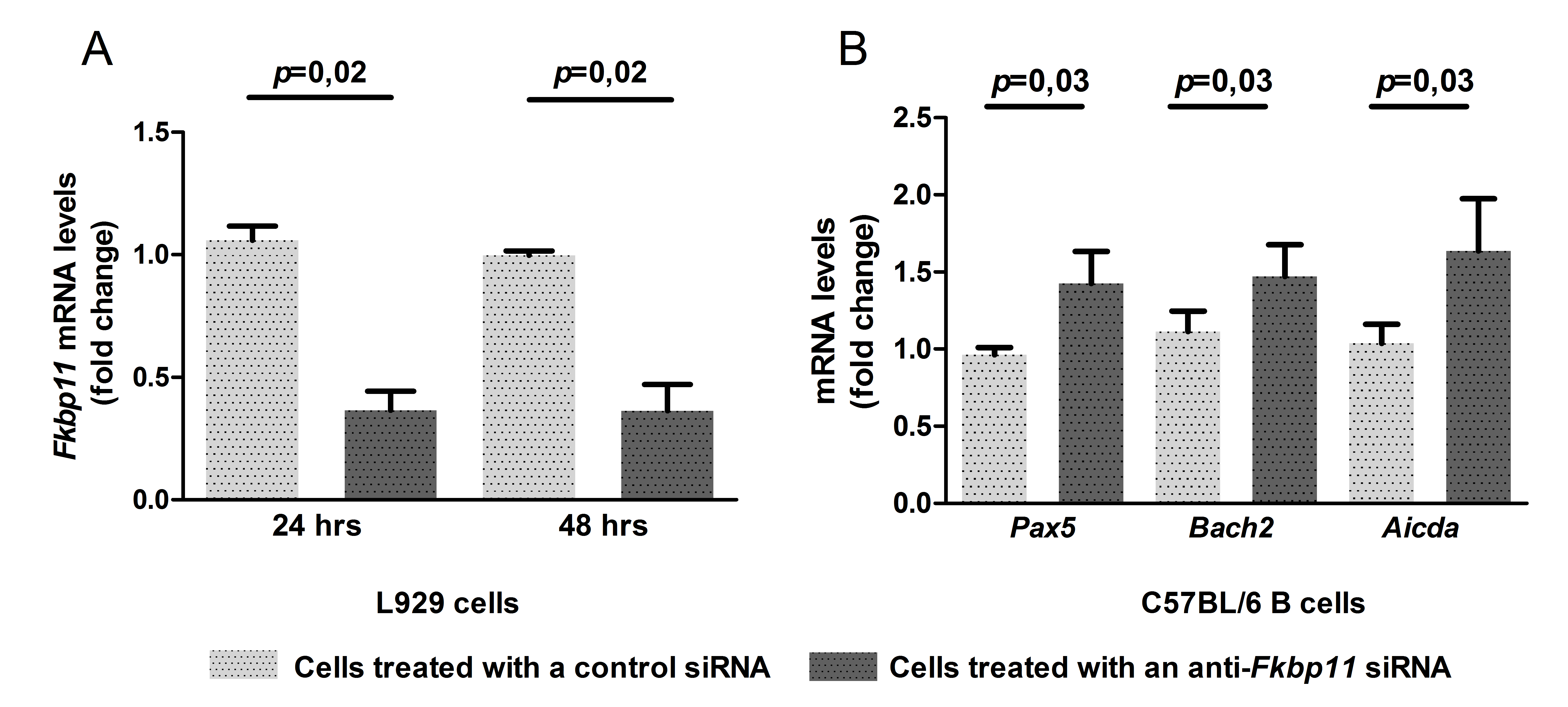
**

**Supplementary Fig. 5. Increased expression of *Pax5*, *Bach2* and *Aid* in *Fkbp11* knock-down B cells after PB differentiation’s induction.**

(**A**) Efficiency of anti-*Fkbp11* siRNA was tested in L929 murine fibroblasts, by real-time quantitative RT-PCR analysis of *Fkbp11* expression, 24h and 48h after incubation with an anti-*Fkbp11* or a control siRNA. Each sample was normalized to the endogenous control *Hprt1* (*n*=3 for each group; *p*: Wilcoxon match-pairs test) (Error bars, SEM). (**B**) Expression of master genes involved in plasma cell differentiation in purified splenic mature B cells from 3-month-old C57BL/6 mice, treated with anti-*Fkbp11* or control siRNA, then stimulated with LPS for 4 days *in vitro*. RNA was extracted, then quantitative real time RT-PCR analysis of mRNA expression was performed for the indicated genes. Each sample was normalized to the endogenous control *Hprt1*. (*n*=6 for each group; *p*: Wilcoxon match-pairs test) (Error bars, SEM).
